# Temporal, spatial and demographic distributions characteristics of COVID-19 symptom clusters from chinese medicine perspective: a systematic cross-sectional study in China from 2019 to 2023

**DOI:** 10.1186/s13020-024-01043-4

**Published:** 2024-12-18

**Authors:** Bin Liu, Tian Song, Mingzhi Hu, Zhaoyuan Gong, Qianzi Che, Jing Guo, Lin Chen, Haili Zhang, Huizhi Li, Ning Liang, Jing Wan, Kunfeng Wang, Yanping Wang, Nannan Shi, Luqi Huang

**Affiliations:** 1 Institute of Basic Research in Clinical Medicine, China Academy of Chinese Medical Sciences, Dongcheng District, Beijing, 100700 China; 2 College of Information Science and Technology, Beijing University of Chemical Technology, Chaoyang District, Beijing, 100029 China; 3 China Academy of Chinese Medical Sciences, Dongcheng District, Beijing, 100700 China

**Keywords:** Chinese medicine, Symptom cluster, Treatment in accordance with three categories of etiologic factors, Subtypes diagnosis, COVID-19

## Abstract

**Background:**

The subtypes diagnosis of disease symptom clusters, grounded in the theory of “Treatment in Accordance with Three Categories of Etiologic Factors” and International Classification of Diseases 11th Revision (ICD-11), is a vital strategy for Chinese Medicine (CM) in treating unknown respiratory infectious diseases. However, the classification of disease symptom clusters continues to depend on empirical observations and lacks robust scientific evidence. Consequently, this study seeks to explore the temporal, spatial and demographic distributions characteristics of Corona Virus Disease 2019 (COVID-19) symptom clusters in China.

**Methods:**

PubMed, Web of Science, Science direct, WHO, Litcovid, CNKI databases were searched from inception until December 31, 2023. Optical character recognition technology and image recognition technology were employed to identify tables within the papers. Four researchers independently screened and extracted data, resolving conflicts through discussion. Heat mapping and hierarchical clustering techniques were utilized to analyze COVID-19 symptom clusters. Data analysis and visualization were conducted using R software (4.2.0), while the association analysis of symptom clusters was performed using Cytoscape (3.10.2).

**Results:**

A total of 366 COVID-19 clinical trials with 86,972 cases including 66 clinical symptoms of 7 disease systems and other clinical manifestations in China were included. In temporal distribution, 63 symptoms centered around fatigue and 44 symptoms focused on chest tightness are characteristic of symptom clusters in spring and winter, respectively. With the addition of spatial distribution, the symptom clusters in middle and low latitudes during spring are characterized by 53 symptoms centered around fatigue and cough, and 51 symptoms focused on fatigue, respectively. During winter, the symptom clusters in middle and low latitudes are characterized by 38 symptoms centered around chest tightness and 37 symptoms focused on fever, respectively. When considering demographic distribution, the symptom clusters for < 50 years are characterized by fatigue as the core symptom in middle (44 symptoms)/low (28 symptoms) latitudes during spring and middle latitude (25 symptoms) during winter. For ≥ 50 years, the symptom clusters in middle latitude (49 symptoms) during spring and low latitudes (35 symptoms) during winter are centered around cough, while in low latitude (27 symptoms) focuses on diarrhea during spring, and middle latitude (35 symptoms) emphasizes both diarrhea and chest tightness during winter.

**Conclusion:**

In summary, variations in symptom clusters and core symptoms of COVID-19 in temporal, spatial and demographic distributions in China offer a scientific rationale for the “Treatment in Accordance with Three Categories of Etiologic Factors” theory. These interesting findings prompt further investigation into CM patterns in the ICD-11, and suggest potential strategies for personalized precision treatment of COVID-19. High-quality clinical studies focusing on individual symptoms are warranted to enhance understanding of respiratory infectious diseases.

**Supplementary Information:**

The online version contains supplementary material available at 10.1186/s13020-024-01043-4.

## Introduction

Having emerged as a new respiratory infectious disease, corona virus disease 2019 (COVID-19) has been causing serious health problems around the world [1]. Based on 3000 years of experience, Chinese medicine (CM) has developed the theory of “Treatment in Accordance with Three Categories of Etiologic Factors”, which is one of the most basic principles of CM treatment, including “treatment in accordance with seasons”, “treatment in accordance with geographic features” and “treatment in accordance with individualized conditions” [2, 3]. It holds significant guiding importance for CM in the treatment of diseases, reflecting the holistic perspective of CM scholars regarding the relationship between nature, humanity, and society [4]. Furthermore, individualized treatment strategies were formulated according to different clinical symptoms, considering the three factors of seasons, geographic features, and individual conditions [5]. Symptom clusters comprising three or more interrelated symptoms that occur simultaneously, regardless of whether they share the same etiological mechanism, serve as the foundation for the diagnosis and classification of CM [6]. Meanwhile, symptom clusters can simplify the components of the pattern and improve the accuracy of diagnosis [7]. In the process of China's response to the COVID-19 epidemic, the diagnosis and classification of traditional medicine based on International Classification of Diseases 11th Revision (ICD-11) [8, 9], and Chinese Pattern Classification and Treatment have played a pivotal role [10].

Clinical symptoms of patients are used in the subtype diagnosis and treatment of CM, and clinical symptoms are also used for judging clinical efficacy. Food and Drug Administration Patient-Focused Drug Development Guidance already uses clinical symptoms as an outcome indicator for the development of new drugs for major diseases [11]. The improvement in clinical symptoms of COVID-19 is one of the most important outcomes of new drug research and development [12]. Previous studies have found that the occurrence of clinical symptoms of COVID-19 will change with time/seasons [13, 14], different geographical locations [15, 16] and different populations [17, 18] present different characteristics.

For example, clinical symptoms may worsen or decrease with the passage of time [13]. Depending on the type of coronavirus strain infected, the clinical symptoms may differ, which reflects the dynamic development of time over time [19–22]. With increasing geographical latitudes, COVID-19’s clinical symptoms increased, and its mortality rate increased as well [16]. Eastern and Western countries also have different clinical symptoms [15]. This provides an inspiration for the spatial–temporal distribution evidence of CM diagnosis of COVID-19. Just as the seasonality of respiratory syncytial virus varies widely among provinces in China, the characteristics of infection and clinical symptoms of COVID-19 should also be seasonal [23]. In particular, both influenza and COVID-19 have emerged during the winter season for the first time [24, 25]. And they usually occur typically during winter and early spring [26]. Additionally, some researchers have begun to pay attention to the seasonality of COVID-19 clinical symptoms and found that symptoms such as cough are related to the season, and that different signs and symptoms should be considered when diagnosing COVID-19 [14]. In adult patients, pediatric patients and elderly patients, the clinical symptoms of COVID-19 are different, especially the younger the age, the milder the clinical symptoms and the better the prognosis [17, 18]. Different racial/ethnicities, genders, and ages had different clusters of symptoms, allowing for more accurate diagnosis of COVID-19 [7, 27]. Pattern differentiation in CM can help reveal specific clinical symptoms to guide biomedical therapy [28].

Although the evidence presented above directly or indirectly supports aspects of the CM theory of “Treatment in Accordance with Three Categories of Etiologic Factors”, there remains a significant gap in research exploring the influence of seasons, geographic features, and individual conditions on the symptoms of respiratory infectious diseases. As a newly emerging respiratory infectious disease with a high incidence in spring and winter, there have been numerous COVID-19 clinical trials since December 2019. These trials not only focus on diagnosis, treatment, and the development of new drugs and vaccines but also place greater emphasis on the statistical analysis of clinical symptoms associated with the novel coronavirus.

Therefore, this study will focus on the clinical research related to COVID-19 conducted in China from December 2019 to December 2023, examining data across the spring and winter seasons, as well as among populations at middle and low latitudes. The aim is to investigate the temporal, spatial, and demographic distributions characteristics of COVID-19 symptom clusters in China. This research seeks to provide scientific support for the theory of “Treatment in Accordance with Three Categories of Etiologic Factors”. Additionally, it aims to propose a new rapid diagnostic strategy for addressing emerging respiratory infectious diseases in the future, thereby enhancing the role of traditional medicine in epidemic prevention and control.

## Materials and methods

### Study design

This study is a systematic cross-sectional analysis that involves a comprehensive search and collection of data from published clinical studies on COVID-19 in China, covering the period from December 2019 to December 2023. First, based on the median duration of clinical trials, we categorized them into spring and winter (Spring: 2020.2.4–2020.5.4, 2021.2.3–2021.5.4, 2022.2.4–2022.5.4, 2023.2.4–2023.5.5; Winter: 2019.12.1–2020.2.3, 2020.11.7–2021.2.2, 2021.11.7–2022.2.3, 2022.11.7–2023.2.3, 2023.11.8–2023.12.31) temporal distribution to conduct a comparative analysis of the characteristics of COVID-19 symptom clusters. Secondly, on the basis of temporal distribution, clinical trial sites were classified into middle latitude (Provinces of China between 30°N and 60°N) and low latitude (Provinces of China between 0°N and 30°N) for a spatial distribution analysis of COVID-19 symptom clusters. Finally, considering both temporal and spatial distributions, we divided patients in the clinical trials into < 50 years old and ≥ 50 years old based on the median/mean age [29, 30]. We then compared and analyzed the characteristics of COVID-19 symptoms across these groups.

### Search strategy

PubMed, Web of Science, Science direct, WHO, Litcovid, CNKI databases were searched from inception until December 31, 2023, without language restrictions for studies to identify published data on COVID-19 clinical symptoms. The following search strategy were used as below: “COVID-19”, “COVID 19”, “2019-nCoV Infection”, “2019 nCoV Infection”, “2019-nCoV Infections”, “Infection, 2019-nCoV”, “SARS-CoV-2 Infection”, “Infection, SARS-CoV-2”, “SARS CoV 2 Infection”, “SARS-CoV-2 Infections”, “2019 Novel Coronavirus Disease”, “2019 Novel Coronavirus Infection”, “2019-nCoV Disease”, “2019 nCoV Disease”, “2019-nCoV Diseases”, “Disease, 2019-nCoV”, “COVID19”, “Coronavirus Disease 2019”, “Disease 2019, Coronavirus”, “Coronavirus Disease-19”, “Coronavirus Disease 19”, “Severe Acute Respiratory Syndrome Coronavirus 2 Infection”, “COVID-19 Virus Disease”, “COVID 19 Virus Disease”, “COVID-19 Virus Diseases”, “Disease, COVID-19 Virus”, “Virus Disease, COVID-19”, “SARS Coronavirus 2 Infection”, “COVID-19 Virus Infection”, “COVID 19 Virus Infection”, “COVID-19 Virus Infections”, “Infection, COVID-19 Virus”, “Virus Infection, COVID-19”, “COVID-19 Pandemic”, “COVID 19 Pandemic”, “Pandemic, COVID-19”, “COVID-19 Pandemics”.

### Selection criteria

Inclusion criteria for clinical experimental studies: (1) Clinical studies must include baseline data and clinical symptom data of COVID-19 patients; any type of clinical trial is acceptable. (2) The clinical trials must have a clearly defined start and end date. (3) The clinical trial must be conducted at a specified site in China. (4) There are no restrictions on the language of the publication. Exclusion Criteria: (1) Studies that do not include clinical symptom data will be excluded. (2) Studies lacking a defined time range for the clinical trial or a specified trial site will be excluded.

### Selection process

PDF files are downloaded based on the deleted entries after we use text similarity calculations to remove duplicate entries from basic information. Optical character recognition technology and image recognition technology were employed to identify tables within the papers and exclude those without COVID-19 clinical symptoms. Four researchers independently screened and extracted data, resolving conflicts through discussion.

### Data collection process

Four researchers (B Liu, T Song, M Hu, and Z Gong) separately imported all the literature into NoteExpress V4.0 software, and after de-duplicating the literature, read the title and abstract, and excluded the studies that did not meet the inclusion exclusion criteria. Studies that may be eligible are then read in full, and the reasons excluded in the screening are recorded in the NoteExpress database. Then, we extracted the following variables from the included studies: (1) Research characteristics: title, study location, study start and end time, study sample size, and study median time. (2) Review specific objectives: Physical health outcomes of SARS-COV-2 infection include: clinical symptoms of respiratory system, nervous system, digestive system, circulatory system, skin system, musculoskeletal system, visual system and other clinical manifestations. In the process of document extraction, if there is any disagreement, it will be resolved through discussion to reach a consensus.

### Data analysis

By calculating the co-occurrence frequency of each pair of symptoms within the same study, we aggregated the discrete symptom data from the literature into a cohesive whole, resulting in the construction of a symmetric matrix. Each element of this matrix represents the number of times the corresponding pair of symptoms co-occurred. Subsequently, the matrix was transformed using the log10 function to compress the data range, allowing for a clearer representation of both high and low frequency co-occurrences in the graph. Finally, we employed heat mapping and hierarchical clustering methods to visualize and categorize the co-occurrence matrix. Data analysis and visualization were conducted using R software (4.2.0). Subsequently, we calculated the correlation coefficients of the clustered symptoms and constructed a comprehensive interaction network using Cytoscape software (version 3.10.2). We focused on the core symptoms that exhibit the most connections with other clinical symptoms, using “degree” as the measure of connectivity between symptoms.

## Results

### Characteristics of COVID-19 clinical symptoms included in China

**Fig. 1:**
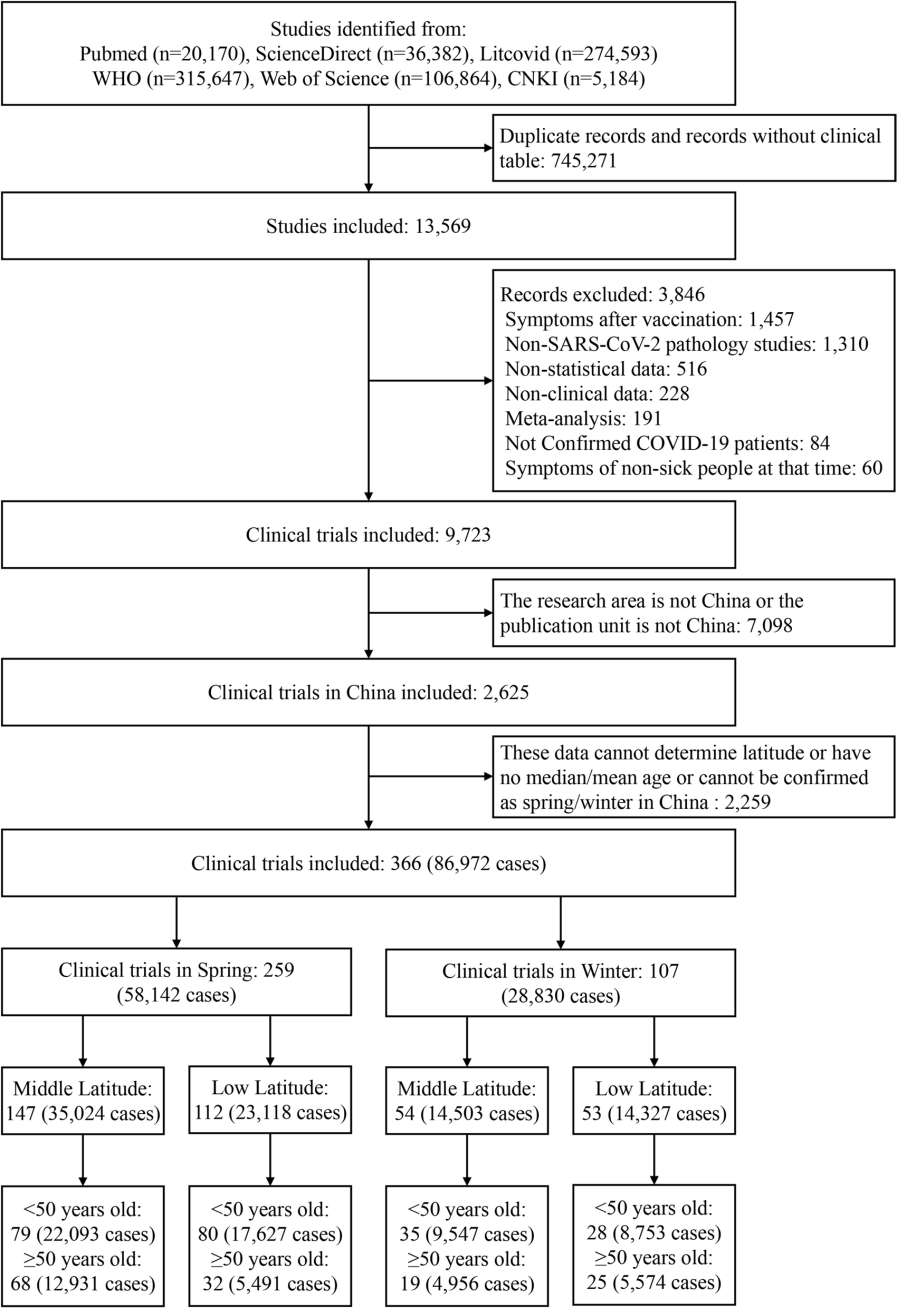
Flowchart of COVID-19 clinical trials and cases were included in China

A total of 13,569 records have been identified. After the exclusion, 9723 records were screened, and subsequently 2625 full text reports were assessed for eligibility, of which 366 COVID-19 clinical trials with 86,972 cases in China met the eligibility criteria. Among them, 259 clinical trials conducted in spring included a total of 58,142 cases, while 107 clinical trials conducted in winter involved 28,830 cases. In spring, there were 147 middle latitude clinical trials that encompassed 35,024 cases, including 79 trials with 22,093 cases < 50 years old and 68 trials with 12,931 cases ≥ 50 years old. Additionally, there were 112 low latitude clinical trials in spring, involving 23,118 cases, which included 80 trials for individuals < 50 years of age (17,627 cases) and 32 trials for those ≥ 50 years of age (5491 cases). In winter, 54 middle latitude clinical trials included 14,503 cases, consisting of 35 trials with 9547 cases < 50 years old and 19 trials with 4956 cases ≥ 50 years old. Furthermore, there were 53 low latitude clinical trials in winter, involving 14,327 cases, which included 28 trials with 8753 cases < 50 years and 25 trials with 5574 cases ≥ 50 years (Fig. 1). The comparison revealed that the number of clinical trials and cases ≥ 50 years was lower than that of those < 50 years in both middle latitude and low latitude regions during spring and winter. Summary information on the included studies and datasets is provided in Table S1.

### Symptom cluster characteristics of COVID-19 during spring and winter seasons in China

**Fig. 2:**
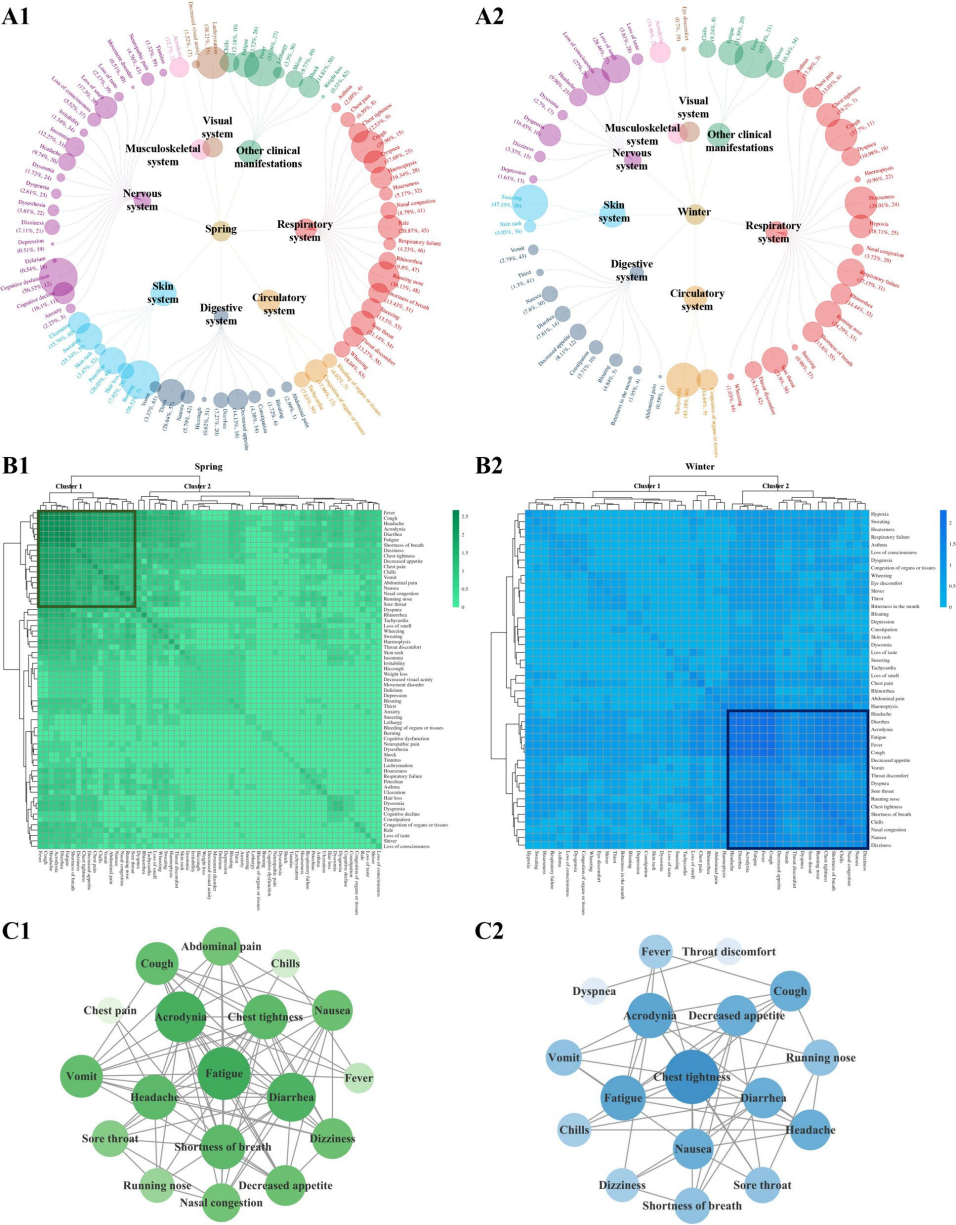
Characteristics of COVID-19 symptom clusters during spring and winter seasons in China. **A1** A total of 63 clinical symptoms were distributed in 7 disease systems and other clinical manifestations of COVID-19 in spring; **B1** Cluster 1 was identified as the core symptom cluster in spring, with varying shades of green indicating the frequency of co-occurring symptoms; **C1** Symptom correlation analysis showed fatigue was the key symptom in spring, and both the shade of green and the size of the circle represented the frequency of association between symptoms; **A2** A total of 44 clinical symptoms were distributed in 7 disease systems and other clinical manifestations of COVID-19 in winter; **B2** Cluster 2 was identified as the core symptom cluster in winter, with varying shades of blue indicating the frequency of co-occurring symptoms; **C2** Symptom correlation analysis showed chest tightness was the key symptom in winter, and both the shade of blue and the size of the circle represented the frequency of association between symptoms

In spring, a total of 63 clinical symptoms were identified across 7 disease systems and other clinical manifestations (Fig. 2A1), representing an increase from the 44 clinical symptoms recorded in winter from the same 7 disease systems and other clinical manifestations (Fig. 2A2). The incidence of respiratory, nervous and digestive symptoms was the highest in spring and winter seasons. Beyond these, the incidence rates for the circulatory system, musculoskeletal system, and other clinical manifestations were comparable in both seasons. Notably, the incidence of skin-related symptoms in winter (38.87%) was higher than in spring (21.15%), while the incidence of visual system symptoms was lower in winter (0.7%) compared to spring (23.69%) (Fig. S1).

The results of the symptom co-occurrence matrix and hierarchical cluster analysis indicated that both spring (Fig. 2B1) and winter (Fig. 2B2) feature a high-frequency core symptom cluster. Among the more prevalent clinical symptoms, acrodynia was reported in 12.70% of cases during spring, compared to 14.46% in winter. Other symptoms included: chest tightness (12.53% in spring vs. 19.20% in winter), chills (12.18% in spring vs. 9.24% in winter), cough (39.96% in spring vs. 37.70% in winter), decreased appetite (14.13% in spring vs. 8.11% in winter), diarrhea (7.27% in spring vs. 7.61% in winter), dizziness (7.11% in spring vs. 3.37% in winter), fatigue (23.72% in spring vs. 21.39% in winter), fever (53.09% in spring vs. 52.74% in winter), headache (9.74% in spring vs. 9.98% in winter), nasal congestion (8.79% in spring vs. 3.72% in winter), nausea (5.79% in spring vs. 7.80% in winter), running nose (36.13% in spring vs. 24.29% in winter), shortness of breath (13.43% in spring vs. 13.60% in winter), sore throat (21.14% in spring vs. 23.90% in winter), and vomit (3.37% in spring vs. 2.79% in winter). In addition, abdominal pain (2.99%) and chest pain (6.59%) were also reported in spring, and dyspnea (10.98%) and throat discomfort (9.24%) were reported in winter. While most symptoms in the core symptom clusters for both seasons are similar, the correlation analysis reveals that fatigue exhibits the highest number of connections with other symptoms in spring, totaling 15. This makes it the key symptom of the spring core symptom cluster (Fig. 2C1). In winter, chest tightness, along with other symptoms, had the highest number of associations, also totaling 13, thereby establishing it as the key symptom of the winter core symptom cluster (Fig. 2C2).

In addition, we analyzed the characteristics of COVID-19 symptom clusters based on middle and low latitudes, as well as age groups above and below 50 years. The results indicated that there were 56 clinical symptoms of COVID-19 identified in middle latitude (Fig. S2A1) and 58 in low latitude (Fig. S2A2). The incidence of symptoms common to both regions was similar. In the symptom co-occurrence matrix and hierarchical cluster analysis, both the middle latitude (Fig. S2B1) and low latitude (Fig. S2B2) exhibited a high-frequency core symptom cluster. Correlation analysis revealed that running nose had the highest number of connections with other symptoms in the middle latitude, totaling 11 associations. This positions them as key symptom within the middle latitude core symptom cluster (Fig. S2C1). In the low latitude, diarrhea exhibited the highest number of associations, also totaling 9, thereby establishing it as the key symptom of the low latitude core symptom cluster (Fig. S2C2). When analyzing by age, patients ≥ 50 years exhibited 62 symptoms (Fig. S3A2), while those younger than 50 years showed 47 symptoms (Fig. S3A1). Although patients ≥ 50 years had a greater variety of clinical symptoms, the number of symptoms in their high-frequency core symptom cluster (Fig. S2B2) was lower than that of patients < 50 years (Fig. S2B1). Correlation analysis indicated that nausea had the highest number of connections with other symptoms in patients < 50 years, totaling 9 degrees, making it the key symptom of the middle latitude core symptom cluster (Fig. S3C1). For patients ≥ 50 years, chest tightness had the highest number of associations, totaling 11, thereby establishing it as the key symptom of the low latitude core symptom cluster (Fig. S3C2).

Overall, the characteristics of COVID-19 symptom clusters varied significantly when analyzed by season, latitude, and age. While chest tightness was identified as a core symptom for both patients ≥ 50 years and during winter, the core symptoms in other dimensions differed. This suggests the need for a comprehensive analysis considering temporal, spatial and demographic distributions. Therefore, the next step will be to incorporate middle and low latitudes for in-depth analysis in the context of spring and winter.

### Symptom cluster characteristics of COVID-19 in middle and low latitudes during spring and winter in China

**Fig. 3:**
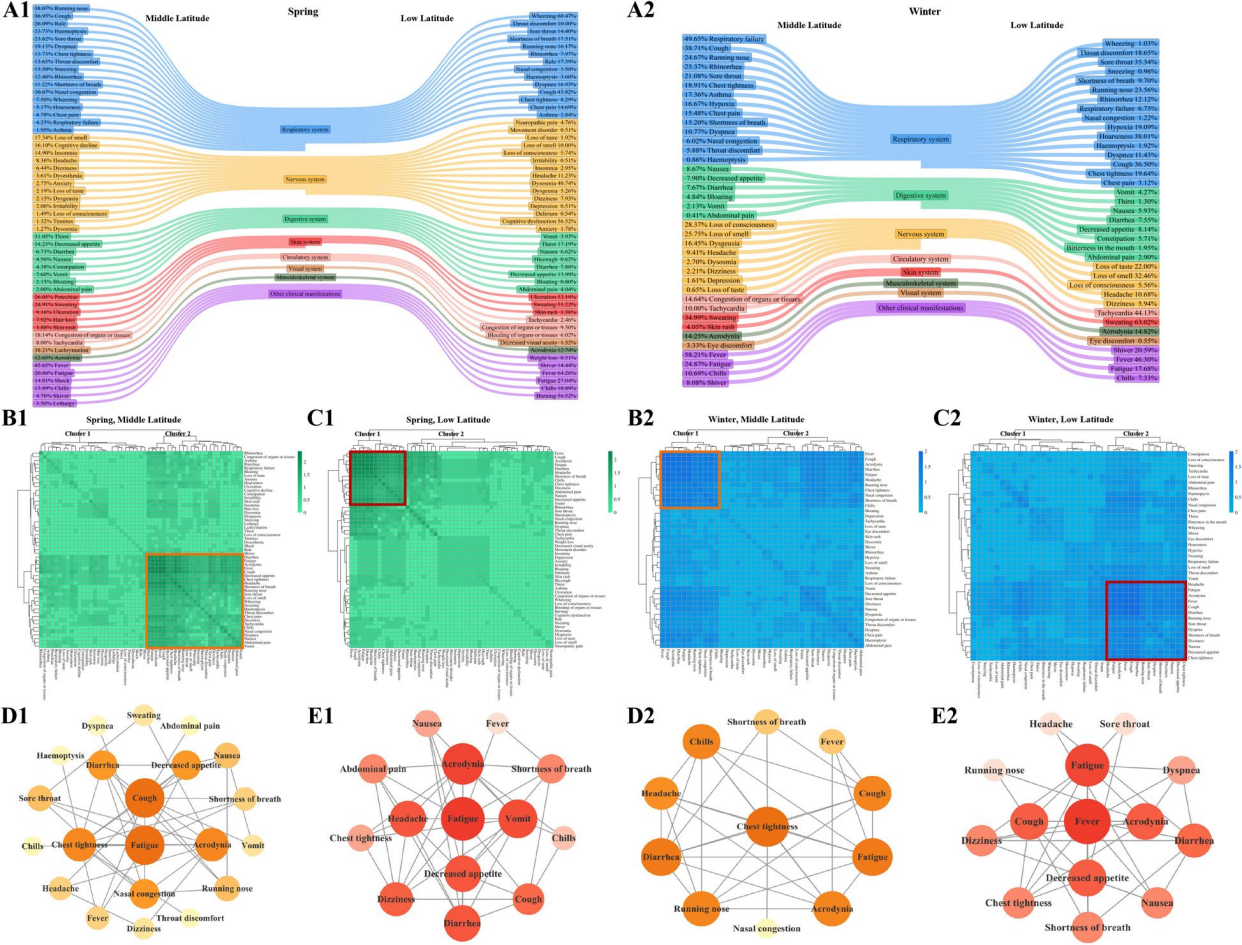
Characteristics of COVID-19 symptom clusters in middle and low latitudes during spring and winter seasons in China. **A1** During spring, there were 53 and 51 clinical symptoms were distributed in 7 disease systems and other clinical manifestations of COVID-19 in middle latitude and low latitude, respectively; **B1**, **C1** Cluster 2 and Cluster 1 were identified as the COVID-19 core symptom cluster in middle latitude and low latitude regions during spring, respectively. Varying shades of green indicating the frequency of co-occurring symptoms; **D1** Symptom correlation analysis showed fatigue and cough were the key symptoms in middle latitude during spring, and both the shade of yellow and the size of the circle represented the frequency of association between symptoms; **E1** Symptom correlation analysis showed fatigue was the key symptoms in low latitude during spring, and both the shade of red and the size of the circle represented the frequency of association between symptoms; **A2** During winter, there were 38 and 37 clinical symptoms were distributed in 7 disease systems and other clinical manifestations of COVID-19 in middle latitude and low latitude, respectively; **B2, C2** Cluster 1 and Cluster 2 were identified as the COVID-19 core symptom cluster in middle latitude and low latitude regions during winter, respectively. Varying shades of blue indicating the frequency of co-occurring symptoms; **D2** Symptom correlation analysis showed chest tightness was the key symptoms in middle latitude during winter, and both the shade of yellow and the size of the circle represented the frequency of association between symptoms; **E2** Symptom correlation analysis showed fever was the key symptoms in low latitude during winter, and both the shade of red and the size of the circle represented the frequency of association between symptoms

In spring and winter, the number of clinical symptoms in middle latitude was higher than that in low latitude. There were 53 and 51 clinical symptoms of 7 disease systems and other clinical manifestations in middle latitude and low latitude regions during spring, respectively (Fig. 3A1). There were 38 and 37 clinical symptoms of 7 disease systems and other clinical manifestations in middle latitude and low latitude regions during winter, respectively (Fig. 3A2). In both seasons, there was a higher incidence of skin system symptoms and a lower incidence of visual system symptoms in low latitude than in middle latitude. Interestingly, circulatory symptoms were more common in middle latitude during spring (12.29% in middle latitude vs. 4.26% in low latitude). In winter, circulatory symptoms were more common in low latitude (14.61% in middle latitude vs. 44.13% in low latitude) (Fig. S4).

The results of the symptom co-occurrence matrix and hierarchical cluster analysis indicated that in spring, the number of core symptom clusters was higher in middle latitude (Fig. 3B1) than in low latitude (Fig. 3C1). The symptoms of middle and low latitudes core symptom clusters are: abdominal pain (2.00% in middle latitude vs. 4.04% in low latitude), acrodynia (12.65% in middle latitude vs. 12.74% in low latitude), chest tightness (13.73% in middle latitude vs. 8.29% in low latitude), chills (13.89% in middle latitude vs. 10.89% in low latitude), cough (36.95% in middle latitude vs. 43.82% in low latitude), decreased appetite (14.25% in middle latitude vs. 13.99% in low latitude), diarrhea (6.73% in middle latitude vs. 7.88% in low latitude), dizziness (6.44% in middle latitude vs. 7.93% in low latitude), fatigue (20.86% in middle latitude vs. 27.04% in low latitude), fever (45.65% in middle latitude vs. 64.26% in low latitude), headache (8.36% in middle latitude vs. 11.23% in low latitude), nausea (4.56% in middle latitude vs. 6.62% in low latitude), shortness of breath (11.22% in middle latitude vs. 17.51% in low latitude), vomit (2.60% in middle latitude vs. 3.93% in low latitude). Middle latitude also has symptoms like chest pain (4.78%), dyspnea (19.13%), haemoptysis (23.73%), loss of smell (17.34%), nasal congestion (10.67%), running nose (38.07%), sore throat (23.62%), sweating (24.91%), tachycardia (8.00%), throat discomfort (13.63%), wheezing (7.50%). The results of correlation analysis showed that fatigue and cough, as key symptoms, were most associated with other symptoms (both 11 degrees) in middle latitude region during spring (Fig. 3D1). As a key symptom, fatigue in low latitude areas during spring had the most associations with other symptoms (11 degrees) (Fig. 3E1).

The results of the symptom co-occurrence matrix and hierarchical cluster analysis indicated that during winter, the number of core symptom clusters was lower in middle latitude (Fig. 3B2) than in low latitude (Fig. 3C2). The symptoms of middle and low latitude core symptom clusters are: acrodynia (14.25% in middle latitude vs. 14.82% in low latitude), chest tightness (18.91% in middle latitude vs. 19.64% in low latitude), cough (38.71% in middle latitude vs. 36.50% in low latitude), diarrhea (7.67% in middle latitude vs. 7.55% in low latitude), fatigue (24.87% in middle latitude vs. 17.68% in low latitude), fever (58.21% in middle latitude vs. 46.30% in low latitude), headache (9.41% in middle latitude vs. 10.68% in low latitude), running nose (24.67% in middle latitude vs. 23.56% in low latitude), shortness of breath (15.20% in middle latitude vs. 9.70% in low latitude). Middle latitude has chills (10.69%), nasal congestion (6.02%). And low latitude has decreased appetite (8.14%), dizziness (5.94%), dyspnea (11.43%), nausea (5.93%), sore throat (35.34%). Correlation analysis results showed that chest tightness, as a key symptom, was most associated with other symptoms in middle latitude during winter (9 degrees) (Fig. 3D2). In winter, fever at low latitude, as a key symptom, was most associated with 10 other symptoms (Fig. 3E2).

### COVID-19 symptom cluster characteristics of patients under and over 50 years of age in middle and low latitudes during spring

**Fig. 4:**
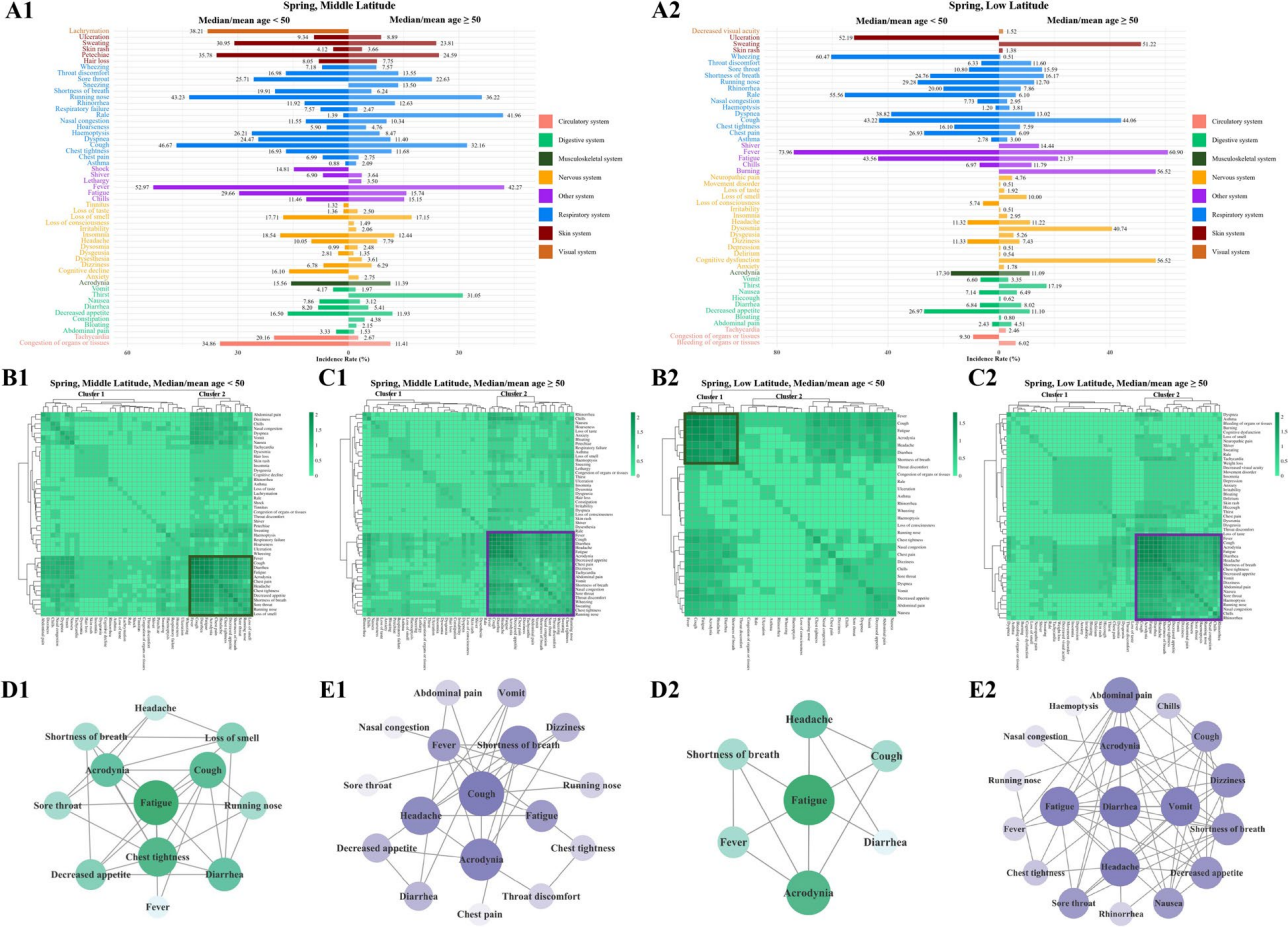
Characteristics of COVID-19 symptom clusters in patients under and over 50 years of age in middle and low latitudes during spring in China. **A1** In middle latitude, there were 44 and 49 clinical symptoms were distributed in COVID-19 patients < 50 years and ≥ 50 years, respectively; **B1**, **C1** Two Cluster 2 were identified as the COVID-19 core symptom cluster in patients < 50 years and ≥ 50 years in middle latitude, respectively. Varying shades of green indicating the frequency of co-occurring symptoms; **D1** Symptom correlation analysis showed fatigue was the key symptoms in patients < 50 years in middle latitude, and both the shade of green and the size of the circle represented the frequency of association between symptoms; **E1** Symptom correlation analysis showed cough was the key symptoms in patients ≥ 50 years in middle latitude, and both the shade of purple and the size of the circle represented the frequency of association between symptoms; **A2** In low latitude, there were 28 and 47 clinical symptoms were distributed in COVID-19 patients < 50 years and ≥ 50 years, respectively; **B2**, **C2** Cluster 1 and Cluster 2 were identified as the COVID-19 core symptom cluster in patients < 50 years and ≥ 50 years in low latitude, respectively. Varying shades of green indicating the frequency of co-occurring symptoms; **D2** Symptom correlation analysis showed fatigue was the key symptoms in patients < 50 years in low latitude, and both the shade of green and the size of the circle represented the frequency of association between symptoms; **E2** Symptom correlation analysis showed diarrhea was the key symptoms in patients ≥ 50 years in low latitude, and both the shade of purple and the size of the circle represented the frequency of association between symptoms

In middle and low latitudes during spring, the number of clinical symptoms in patients ≥ 50 years was higher than that in patients < 50 years. Patients under and over 50 years of age in middle latitude had 44 and 49 clinical symptoms, respectively (Fig. 4A1). Patients under and over 50 years of age in low latitude had 28 and 47 clinical symptoms, respectively (Fig. 4A2). In low and middle latitudes, patients < 50 years of age have a higher incidence of most systemic disease symptoms than patients ≥ 50 years of age. Especially in the respiratory, digestive, and circulatory systems (Fig. S5A, B).

The results of the symptom co-occurrence matrix and hierarchical cluster analysis indicated that in middle latitude during spring, the number of core symptom clusters in patients < 50 years (Fig. 4B1) is lower than that in patients ≥ 50 years (Fig. 4C1). The symptoms in the core symptom cluster of patients under and above 50 years old are as follows: acrodynia (15.56% in patients < 50 years vs. 11.39% in patients ≥ 50 years), chest pain (6.99% in patients < 50 years vs. 2.75% in patients ≥ 50 years), chest tightness (16.93% in patients < 50 years vs. 11.68% in patients ≥ 50 years), cough (46.67% in patients < 50 years vs. 32.16% in patients ≥ 50 years), decreased appetite (16.50% in patients < 50 years vs. 11.93% in patients ≥ 50 years), diarrhea (8.20% in patients < 50 years vs. 5.41% in patients ≥ 50 years), fatigue (29.66% in patients < 50 years vs. 15.74% in patients ≥ 50 years), fever (52.97% in patients < 50 years vs. 42.27% in patients ≥ 50 years), headache (10.05% in patients < 50 years vs. 7.79% in patients ≥ 50 years), running nose (43.23% in patients < 50 years vs. 36.22% in patients ≥ 50 years), shortness of breath (19.91% in patients < 50 years vs. 6.24% in patients ≥ 50 years), sore throat (25.71% in patients < 50 years vs. 22.63% in patients ≥ 50 years). Patients under the age of 50 had loss of smell (17.71%) and patients over the age of 50 had abdominal pain (1.53%), dizziness (6.29%), nasal congestion (10.34%), tachycardia (2.67%), throat discomfort (13.55%), vomit (1.97%), wheezing (7.57%). The results of correlation analysis showed that fatigue as a key symptom was most frequently associated with other symptoms (10 degrees) in patients < 50 years of age in middle latitude during spring (Fig. 4D1). In patients ≥ 50 years of age, cough was the most associated with other symptoms (8 degrees) as a key symptom (Fig. 4E1).

The results of the symptom co-occurrence matrix and hierarchical cluster analysis indicated that in low latitude during spring, The number of core symptom clusters in patients < 50 years (Fig. 4B2) is lower than that in patients ≥ 50 years (Fig. 4C2). The core symptom clusters in patients under 50 years old and above all have the following symptoms: acrodynia (17.30% in patients < 50 years vs. 11.09% in patients ≥ 50 years), cough (43.22% in patients < 50 years vs. 44.06% in patients ≥ 50 years), diarrhea (6.84% in patients < 50 years vs. 8.02% in patients ≥ 50 years), fatigue (43.56% in patients < 50 years vs. 21.37% in patients ≥ 50 years), fever (73.96% in patients < 50 years vs. 60.90% in patients ≥ 50 years), headache (11.32% in patients < 50 years vs. 11.22% in patients ≥ 50 years), shortness of breath (24.76% in patients < 50 years vs. 16.17% in patients ≥ 50 years). And patients over the age of 50 also had abdominal pain (4.51%), chest tightness (7.59%), chills (11.79%), decreased appetite (11.10%), dizziness (7.43%), haemoptysis (3.81%), nasal congestion (2.95%), nausea (6.49%), rhinorrhea (7.86%), running nose (12.70%), sore throat (15.59%), vomit (3.35%). The results of correlation analysis showed that fatigue, as a key symptom, was most associated with other symptoms (6 degrees) in patients under 50 years of age in low latitude during spring (Fig. 4D2). In patients over 50 years of age, diarrhea, as a key symptom, had the highest number (13 degrees) of associations with other symptoms (Fig. 4E2).

### COVID-19 symptom cluster characteristics of patients under and over 50 years of age in middle and low latitudes during winter

**Fig. 5:**
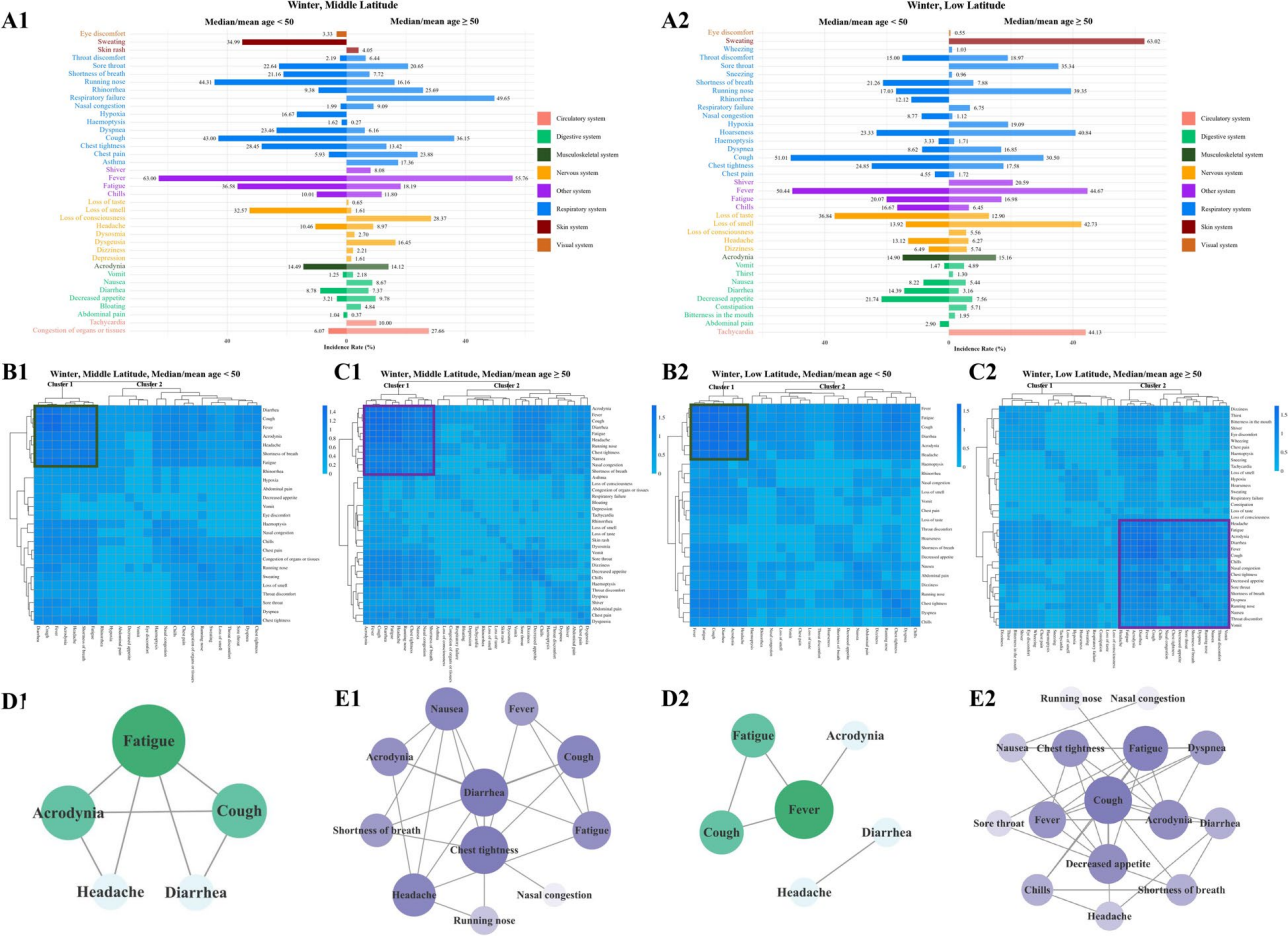
Characteristics of COVID-19 symptom clusters in patients under and over 50 years of age in middle and low latitudes during winter in China. **A1** In middle latitude, there were 25 and 35 clinical symptoms were distributed in COVID-19 patients < 50 years and ≥ 50 years, respectively; **B1**, **C1** Two Cluster 1 were identified as the COVID-19 core symptom cluster in patients < 50 years and ≥ 50 years in middle latitude, respectively. Varying shades of blue indicating the frequency of co-occurring symptoms; **D1** Symptom correlation analysis showed fatigue was the key symptoms in patients < 50 years in middle latitude, and both the shade of green and the size of the circle represented the frequency of association between symptoms; **E1** Symptom correlation analysis showed chest tightness and diarrhea were the key symptoms in patients ≥ 50 years in middle latitude, and both the shade of purple and the size of the circle represented the frequency of association between symptoms; **A2** In low latitude, there were 24 and 35 clinical symptoms were distributed in COVID-19 patients < 50 years and ≥ 50 years, respectively; **B2**, **C2** Cluster 1 and Cluster 2 were identified as the COVID-19 core symptom cluster in patients < 50 years and ≥ 50 years in low latitude, respectively. Varying shades of blue indicating the frequency of co-occurring symptoms; **D2** Symptom correlation analysis showed fever was the key symptoms in patients < 50 years in low latitude, and both the shade of green and the size of the circle represented the frequency of association between symptoms; **E2** Symptom correlation analysis showed cough was the key symptoms in patients ≥ 50 years in low latitude, and both the shade of purple and the size of the circle represented the frequency of association between symptoms

In middle and low latitudes during winter, the number of clinical symptoms in patients ≥ 50 years was more than that in patients < 50 years. Patients under and over 50 years of age in middle latitude had 25 and 35 clinical symptoms, respectively (Fig. 5A1). Patients under and over 50 years of age in low latitude had 24 and 35 clinical symptoms, respectively (Fig. 5A2). In low and middle latitudes, circulatory symptoms were more common in patients older than 50 years and respiratory and digestive symptoms were less common in patients younger than 50 years (Fig. S3C, D).

The results of the symptom co-occurrence matrix and hierarchical cluster analysis indicated that in middle latitude during winter, the number of core symptom clusters in patients under 50 years old (Fig. 5B1) is lower than that in patients over 50 years old (Fig. 5C1). The symptoms in the core symptom cluster of patients under 50 years old and above are: acrodynia (14.49% in patients < 50 years vs. 14.12% in patients ≥ 50 years), cough (43.00% in patients < 50 years vs. 36.15% in patients ≥ 50 years), diarrhea (8.78% in patients < 50 years vs. 7.37% in patients ≥ 50 years), fatigue (36.58% in patients < 50 years vs. 18.19% in patients ≥ 50 years), fever (63.00% in patients < 50 years vs. 55.76% in patients ≥ 50 years), headache (10.46% in patients < 50 years vs. 8.97% in patients ≥ 50 years), shortness of breath (21.16% in patients < 50 years vs. 7.72% in patients ≥ 50 years). And patients over the age of 50 had chest tightness (13.42%), nasal congestion (9.09%), nausea (8.67%), running nose (16.16%). The results of correlation analysis showed that fatigue, as a key symptom, was most associated with other symptoms (4 degrees) in patients under 50 years of age in middle latitude during winter (Fig. 5D1). In patients over 50 years old, chest tightness and diarrhea, as key symptoms, were most associated with other symptoms (6 degrees) (Fig. 5E1).

The results of the symptom co-occurrence matrix and hierarchical cluster analysis indicated that in low latitude during winter, the number of core symptom clusters in patients under 50 years old (Fig. 5B2) was lower than that in patients over 50 years old (Fig. 5C2). The core symptom clusters in patients under 50 years old and over 50 years old were as follows: acrodynia (14.90% in patients < 50 years vs. 15.16% in patients ≥ 50 years), cough (51.01% in patients < 50 years vs. 30.50% in patients ≥ 50 years), diarrhea (14.39% in patients < 50 years vs. 3.16% in patients ≥ 50 years), fatigue (20.07% in patients < 50 years vs. 16.98% in patients ≥ 50 years), fever (50.44% in patients < 50 years vs. 44.67% in patients ≥ 50 years), headache (13.12% in patients < 50 years vs. 6.27% in patients ≥ 50 years). And patients over the age of 50 had chest tightness (17.58%), chills (6.45%), decreased appetite (7.56%), dyspnea (16.85%), nasal congestion (1.12%), nausea (5.44%), running nose (39.35%), shortness of breath (7.88%), sore throat (35.34%), throat discomfort (18.97%), vomit (4.89%). The results of correlation analysis showed that fever, as a key symptom, was the most associated with other symptoms (3 degrees) in patients under 50 years of age at low latitude in winter (Fig. 5D2). In patients over 50 years of age, cough was the most associated with other symptoms (10 degrees) as a key symptom (Fig. 5E2).

## Discussions

**Fig. 6:**
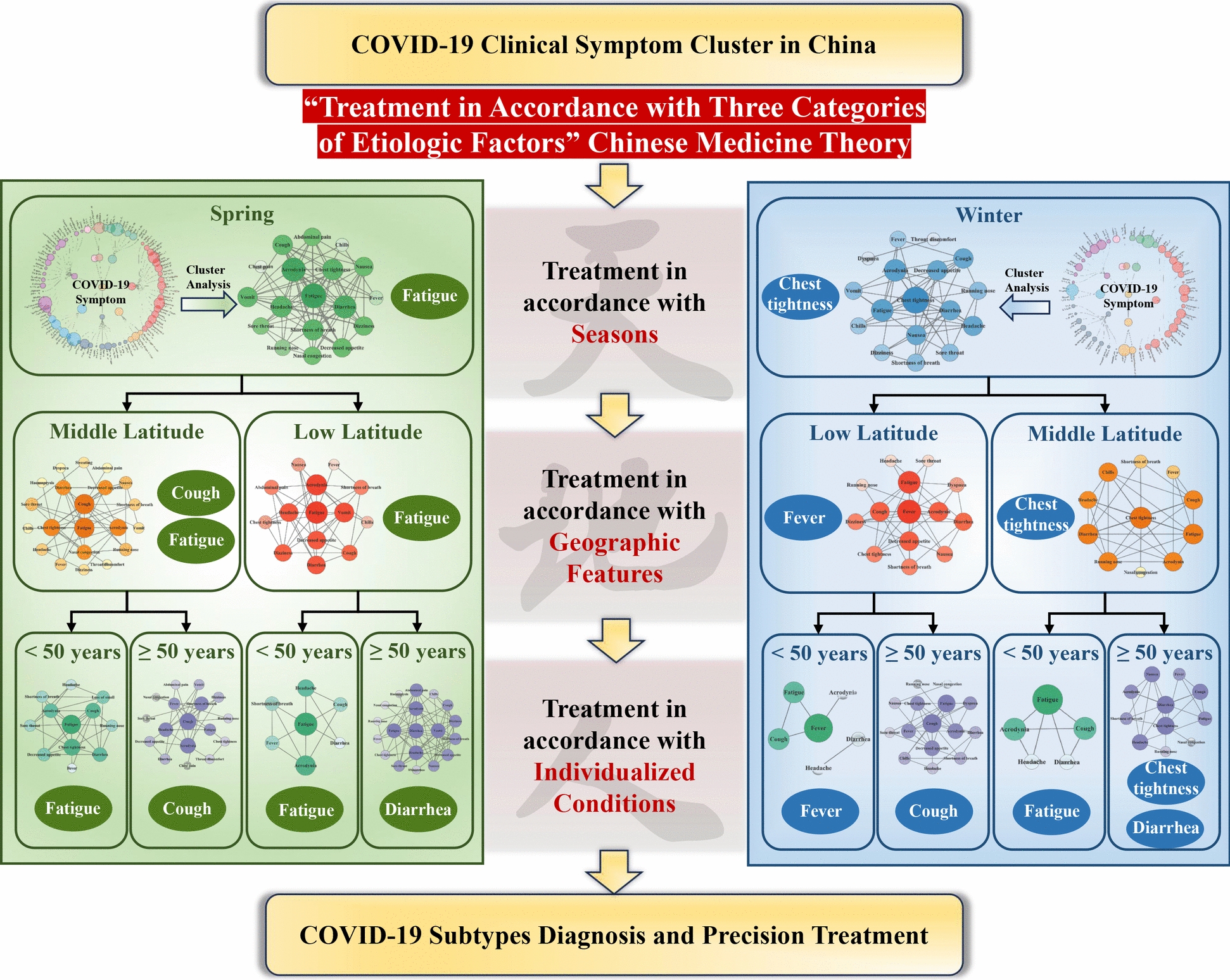
Diagram of seasons, geographic features and individualized characteristics evidence of Chinese Medicine subtypes diagnosis of COVID-19 Symptom Cluster in China from 2019 to 2023

This study comprehensively summarized all COVID-19 clinical trials conducted in China from December 2019 to December 2023, and extracted baseline clinical symptom data of COVID-19 patients in the clinical trials. The characteristics of COVID-19 symptom clusters were analyzed from the temporal distribution (spring, winter), spatial distribution (middle latitude and low latitude) and population distribution (< 50 years and ≥ 50 years). Based on CM theory of “Treatment in Accordance with Three Categories of Etiologic Factors” and the diagnosis classification of traditional medicine based on ICD-11, this study conducted a cluster analysis of COVID-19 symptoms from the perspectives of two seasons, middle/low latitudes and population under/over 50 years, which is a unique diagnosis classification of diseases in CM and provides a basis for the accurate use of Chinese herbal medicine [31, 32]. The results showed that there were differences in the incidence of clinical symptoms, symptom cluster characteristics and core symptoms, which could provide scientific basis for the COVID-19 subtypes diagnosis with CM (Fig. 6).

When focusing exclusively on seasonal factors, the primary symptoms identified in the association analysis were fatigue in spring and chest tightness in winter, despite some similarities between the symptoms of the two seasons. With the warmer temperatures of spring, the body's immune system may become more susceptible to infections from the novel coronavirus, resulting in heightened fatigue [33]. Winter is the season with a high incidence of respiratory viruses. Cold weather can lead to immune system disorders while also causing constriction of the respiratory tract and blood vessels, resulting in an increased sensation of chest tightness [34]. When the range of symptoms analyzed for middle and low latitudes was expanded on a seasonal basis, it was found that the number of symptoms in middle latitude exceeded that in low latitude. Previous studies have shown that adequate sunlight exposure and daily ultraviolet ray doses are linked to lower mortality rates among individuals with COVID-19 [16]. This may be attributed to the fact that as latitude increases, sunlight and ultraviolet radiation decrease, leading to reduced efficiency in the body's synthesis of vitamin D. This vitamin is essential for enhancing immune function and inhibiting excessive inflammatory responses [35]. Moreover, the core symptoms varied: fatigue was prevalent in both middle and low latitudes during spring, while cough was more common in the middle latitudes. As spring temperatures rise, mid-latitude regions tend to be less humid than those at lower latitudes. This dry air can irritate the throat and airways, leading to an increased incidence of cough [36]. In winter, chest tightness emerged as the core symptom in middle latitude regions, whereas fever was the predominant symptom in low latitude areas. Low winter temperatures and fluctuations in humidity at low latitudes can reduce the respiratory tract's resistance. Following infection with the novel coronavirus, the body's immune system is activated, leading to the onset of fever [37].

The introduction of population classifications based on season and geographic region revealed even more intriguing results. Overall, individuals under the age of 50 exhibited fewer symptoms compared to those over 50. Younger individuals typically possess stronger immune systems and are better equipped to respond to viral infections compared to older individuals [38]. As we age, the immune system's function gradually declines, making older individuals more susceptible to infections and experiencing more severe symptoms [39]. Younger individuals' cells may respond more effectively to the virus, facilitating quicker clearance and alleviating symptoms [40]. The primary symptoms for those under 50 were predominantly fatigue in both spring and winter, with fever being the only symptom noted in low latitudes during winter. In contrast, for individuals over 50, cough and diarrhea were the most frequently reported core symptoms in winter and spring, while chest tightness was observed in mid-latitude areas during winter. As people age, their immune systems become less effective, weakening their ability to combat viruses. Additionally, older individuals often have multiple underlying health conditions that increase their susceptibility to infections. Coughing serves as the body's reflex response to respiratory irritation, and older adults may exhibit a heightened nervous system response to such irritation, making them more prone to cough when infected [41]. The novel coronavirus impacts not only the respiratory system but also the digestive system. The digestive systems of older adults tend to be relatively weak and more susceptible to viral infections, leading to symptoms such as diarrhea [42]. These findings provide a foundation for further classification of COVID-19 subtypes and represent an important step toward precision medicine. In diagnosing disease subtypes, CM will consider seasons, geographic features, and individual conditions to optimize the use of proprietary Chinese medicines for treating patients with specific subtypes [43].

The most prevalent symptoms of COVID-19 included fever, cough and fatigue, which also is the main symptom that patients self-reported [13]. Different clinical symptoms of COVID-19 are prevalent at different stages of the pandemic according to the examination of clinical symptom characteristics [44]. The evolution of the variants of severe acute respiratory syndrome coronavirus 2 (SARS-CoV-2) over time is one of the indicators reflecting the different stages of the COVID-19 pandemic. Hosts infected with different variants of SARS-CoV-2 present different clinical symptoms. In Mexico, from 2021 to 2022, anosmia and dysgeusia were mainly found in wild-type SARS-CoV-2 infected patients, while rhinorrhea and sore throat were more prevalent in patients infected with the Omicron variant [45]. In Japan, children infected with different variants also have different clinical symptoms. In the Omicron period, respiratory and gastrointestinal symptoms decreased, and neurological symptoms increased significantly compared to other periods. Altered mental status and seizures were more common during the Omicron period compared to the pre‐Omicron (nonvariant, Alpha, and Delta) period [21]. These evidences show that the clinical symptoms of COVID-19 will change over time, and have a certain relationship with the different variants of COVID-19 strains.

Similarly, differences in spatial distribution will also affect the clinical symptoms of COVID-19. Compared to Western countries, the incidences of cough, headache, dizziness, nasal congestion, and digestive symptoms in COVID-19 patients from the Eastern countries were lower [15]. In Mexico, diarrhea was the main digestive symptom, followed by abdominal pain, and nausea/vomiting. Diarrhea and abdominal pain were more common in West, followed by Southeast and Northwest [46]. Meanwhile, COVID-19 is more severe and has higher death rates in high latitude countries of Europe [16]. This proves from the perspective of different countries that spatial distribution of clinical symptoms can indeed be different.

Different populations display unique clinical characteristics of COVID-19. In comparison to adult patients, pediatric patients show varying laboratory results, milder clinical symptoms, less severe lung radiographic changes, lower classifications of clinical severity, and better clinical outcomes [17]. The percentage and absolute values of lymphocytes, platelet counts, aspartate aminotransferase, and the aspartate aminotransferase/alanine aminotransferase ratio in adults were lower than those in children [47]. Older age, an increased number of comorbidities, and more pronounced abnormal laboratory markers were associated with a more severe condition [48]. Older patients were found to have a higher incidence of severe or critical clinical classifications. In contrast, pediatric patients generally presented either negative chest CT results or involvement of only a single lung lobe. In comparison, older patients demonstrated a greater incidence of involvement across four or five lung lobes [49]. The study revealed that the prevalence of cough in the younger group was negatively correlated with temperature, whereas in the older group, it was negatively correlated with humidity [50]. Furthermore, the incidence of cough varied between the autumn and winter seasons [50].

In the symptom cluster studies, the presence of symptom clusters is potentially associated with the severity of the COVID-19 category [51]. As no single symptom can predict the severity of COVID-19 and the medical support needed, the classification of COVID-19 symptom clusters can be used in the clinic to monitor high-risk patients and predict the need for medical resources days in advance [52]. The study of COVID-19 symptom clusters is considered to replace individual symptom reports, which can guide research into the cause, diagnosis and management of the novel coronavirus after infection [53]. Symptom cluster classification helps distinguish different respiratory diseases in emergency situations and when rapid detection is limited, and is of great significance for improving the development of targeted drugs in the future [54]. Post COVID-19 symptom clusters differ across timeframes. Symptom clusters were useful in establishing coherent patterns of multiple complex symptoms [55]. The discovery of early symptom clusters associated with hospitalization, renewed activity, long-term symptoms, and inflammation may contribute to the prognosis of patients with COVID-19, including predicting long-term COVID risk [56]. By classifying the clinical symptom clusters of intensive care unit (ICU) nurses treating COVID-19 in Wuhan, specific interventions can be implemented to safeguard the health of ICU nurses [57]. This is consistent with the purpose of our research, which is to develop targeted treatment plans for different patients through the classification of COVID-19 symptom clusters, so as to achieve precise treatment of COVID-19. Chinese Pattern Classification and Treatment of COVID-19 is a process of diagnosing and classifying according to the characteristics of symptom clusters and theoretical analysis of CM. This study found that the symptom clusters of COVID-19 were different in different populations in middle/low latitudes during spring and winter seasons in China, which may be due not only to the individual differences in constitution, but also to meteorological factors of temporal and spatial distribution [58].

While our study provides a scientific foundation for CM to diagnose diseases based on seasonal variations, geographic characteristics, and individual symptom groups, it is rooted in clinical data from COVID-19 cases in China. There are still some limitations in this study: (1) Although this study could not obtain a large number of individual data of COVID-19 to analyze the characteristics of symptom clusters. On the one hand, since the COVID-19 epidemic has ended, the number of clinical COVID-19 patients has been greatly reduced, and the toxicity of COVID-19 has been greatly reduced, so it is difficult to obtain detailed data of individuals. On the other hand, although this study used population data on COVID-19, we covered all clinical trials for the entire epidemic period from 2019 to 2023, which has a high degree of confidence and data integrity. The results based on these data analysis not only found some interesting results, but also provided research strategies for the diagnosis and classification of diseases with the characteristics of different spatial, temporal and population symptom clusters; and (2) Given that the seasonal and population classifications were derived from peer-reviewed clinical study data, our analysis utilized both the median duration and median/mean age. Although some studies may explore different seasonal ranges or include age ranges for COVID-19 patients that extend beyond the median or average age, statistical analysis suggests that the median/mean data often provides a more precise and scientifically valid reflection of trends within the majority of data points. This classification led us to uncover intriguing results that provide a robust scientific basis for CM theory. However, specialized multi-regional, multi-center, large-sample clinical trials remain essential to validate the findings of this study.

## Conclusion

In summary, the subtype diagnosis of COVID-19 based on CM theory of “Treatment in Accordance with Three Categories of Etiologic Factors” has temporal, spatial and demographic distributions evidence. It is scientifically possible to treat COVID-19 with Chinese Pattern Classification and Treatment since the symptom cluster characteristics and core symptoms are different. These interesting findings prompt further investigation into CM patterns in the ICD-11, and suggest potential strategies for personalized precision treatment of COVID-19. High-quality clinical studies focusing on individual symptoms are warranted to enhance understanding of respiratory infectious diseases.

## Supplementary Information

Additional file 1. Fig. S1 Distribution characteristics of the disease system with clinical symptoms of COVID-19 in spring and winter.

Additional file 2. Fig. S2 Characteristics of COVID-19 symptom clusters during middle and low latitude in China. (A1) A total of 56 clinical symptoms were distributed in 7 disease systems and other clinical manifestations of COVID-19 in middle latitude; (B1) Cluster 2 was identified as the core symptom cluster in middle latitude, with varying shades of yellow indicating the frequency of co-occurring symptoms; (C1) Symptom correlation analysis showed running nose was the key symptom in middle latitude, and both the shade of yellow and the size of the circle represented the frequency of association between symptoms; (A2) A total of 58 clinical symptoms were distributed in 7 disease systems and other clinical manifestations of COVID-19 in low latitude; (B2) Cluster 1 was identified as the core symptom cluster in low latitude, with varying shades of red indicating the frequency of co-occurring symptoms; (C2) Symptom correlation analysis showed diarrhea was the key symptom in low latitude, and both the shade of red and the size of the circle represented the frequency of association between symptoms.

Additional file 3. Fig. S3 Characteristics of COVID-19 symptom clusters in patients under and over 50 years of age in China. (A1) A total of 47 clinical symptoms were distributed in 7 disease systems and other clinical manifestations of COVID-19 in patients < 50 years; (B1) Cluster 2 was identified as the core symptom cluster in patients < 50 years, with varying shades of green indicating the frequency of co-occurring symptoms; (C1) Symptom correlation analysis showed nausea was the key symptom in patients < 50 years, and both the shade of green and the size of the circle represented the frequency of association between symptoms; (A2) A total of 62 clinical symptoms were distributed in 7 disease systems and other clinical manifestations of COVID-19 in patients ≥ 50 years; (B2) Cluster 1 was identified as the core symptom cluster in patients ≥ 50 years, with varying shades of purple indicating the frequency of co-occurring symptoms; (C2) Symptom correlation analysis showed chest tightness was the key symptom in patients ≥ 50 years, and both the shade of blue and the size of the circle represented the frequency of association between symptoms.

Additional file 4. Fig. S4 Distribution characteristics of the disease system with clinical symptoms of COVID-19 in middle and low latitudes during (A) spring and (B) winter.

Additional file 5. Fig. S5 Distribution characteristics of the disease system with clinical symptoms of COVID-19 in patients < 50 years old and ≥ 50 years old in middle (A)/low (B) latitudes during spring and (B) middle (C)/low (D) latitudes during winter.

Additional file 6. Table S1.

## Data Availability

A paper generating data is included in this article, whereas original data is publicly available. Data extracted from included studies and used for analyses are available on request. According to our search, original articles are available in public databases.

## Abbreviations

ICD-11: International Classification of Diseases 11th Revision
CM: Chinese Medicine
COVID-19: Corona Virus Disease 2019
SARS-CoV-2: Severe acute respiratory syndrome coronavirus 2
