# Supplementary figures and images for: Temporal, spatial and demographic distributions characteristics of COVID-19 symptom clusters from chinese medicine perspective: a systematic cross-sectional study in China from 2019 to 2023
Source: Chin Med. 2024 Dec 18;19:171. doi: 10.1186/s13020-024-01043-4 (PMC11654152; doi:10.1186/s13020-024-01043-4)

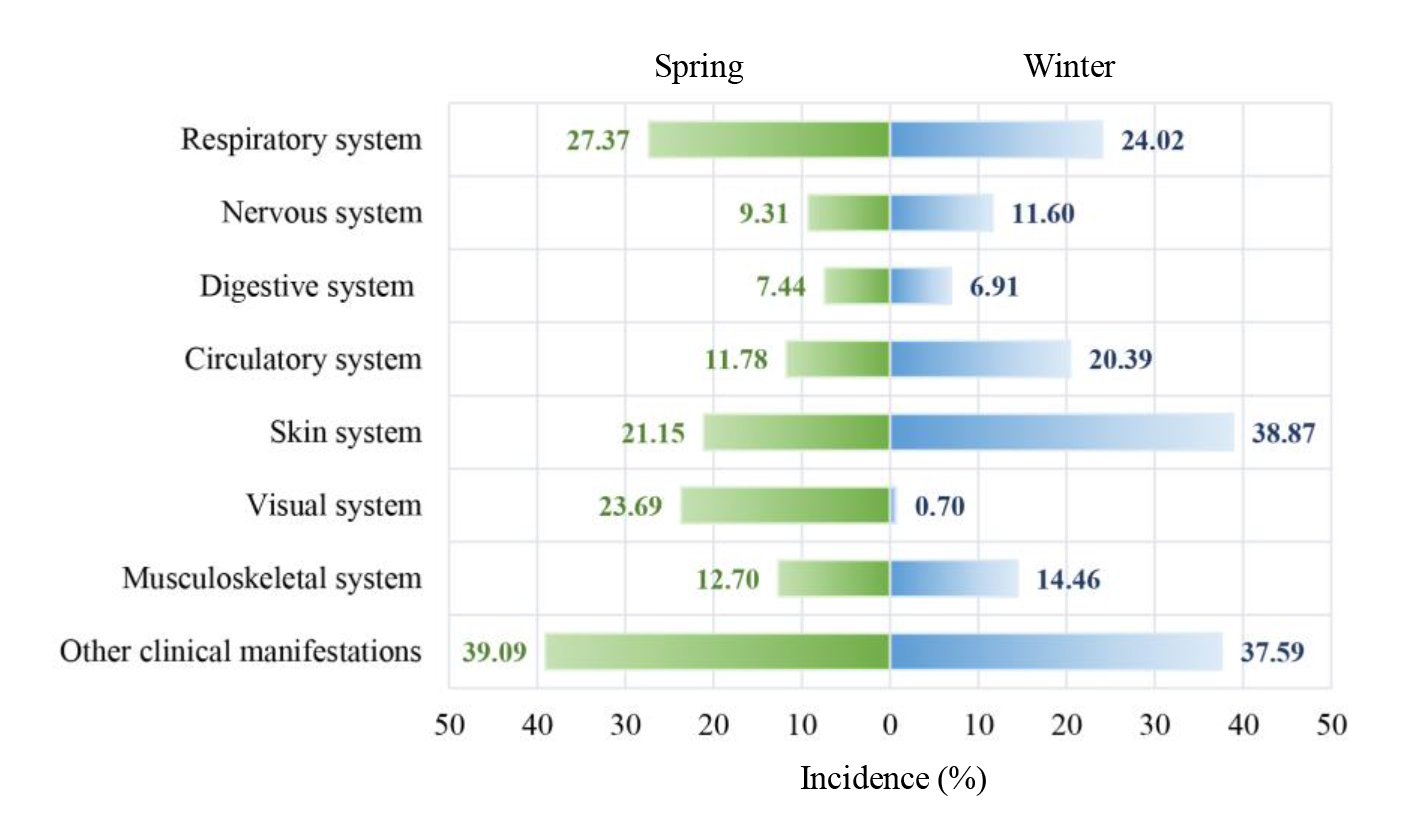

Supplement: Supplementary file 1 — Additional file 1. Fig. S1 Distribution characteristics of the disease system with clinical symptoms of COVID-19 in spring and winter. [file 13020_2024_1043_MOESM1_ESM.jpg]

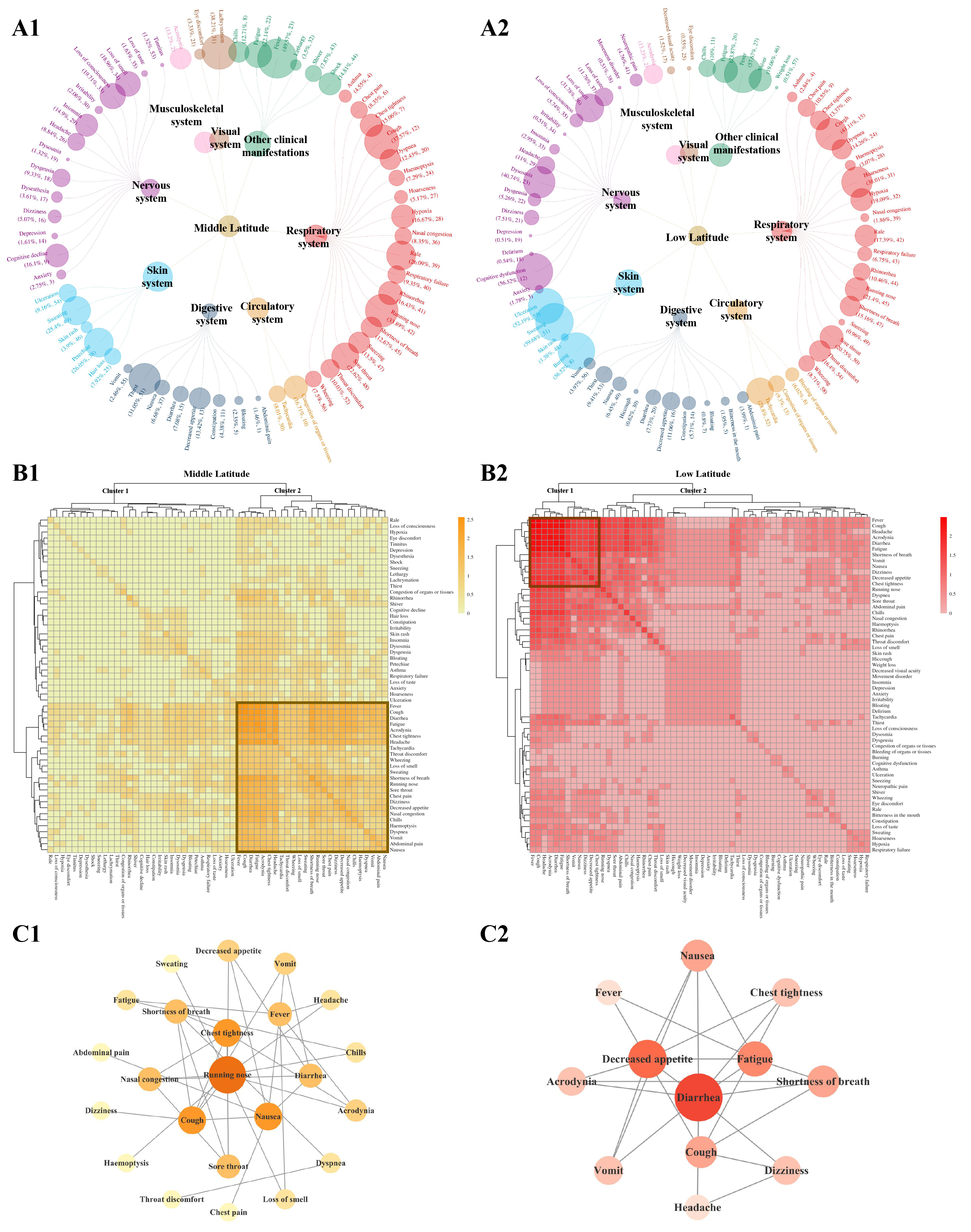

Supplement: Supplementary file 2 — Additional file 2. Fig. S2 Characteristics of COVID-19 symptom clusters during middle and low latitude in China. (A1) A total of 56 clinical symptoms were distributed in 7 disease systems and other clinical manifestations of COVID-19 in middle latitude; (B1) Cluster 2 was identified as the core symptom cluster in middle latitude, with varying shades of yellow indicating the frequency of co-occurring symptoms; (C1) Symptom correlation analysis showed running nose was the key symptom in middle latitude, and both the shade of yellow and the size of the circle represented the frequency of association between symptoms; (A2) A total of 58 clinical symptoms were distributed in 7 disease systems and other clinical manifestations of COVID-19 in low latitude; (B2) Cluster 1 was identified as the core symptom cluster in low latitude, with varying shades of red indicating the frequency of co-occurring symptoms; (C2) Symptom correlation analysis showed diarrhea was the key symptom in low latitude, and both the shade of red and the size of the circle represented the frequency of association between symptoms. [file 13020_2024_1043_MOESM2_ESM.jpg]

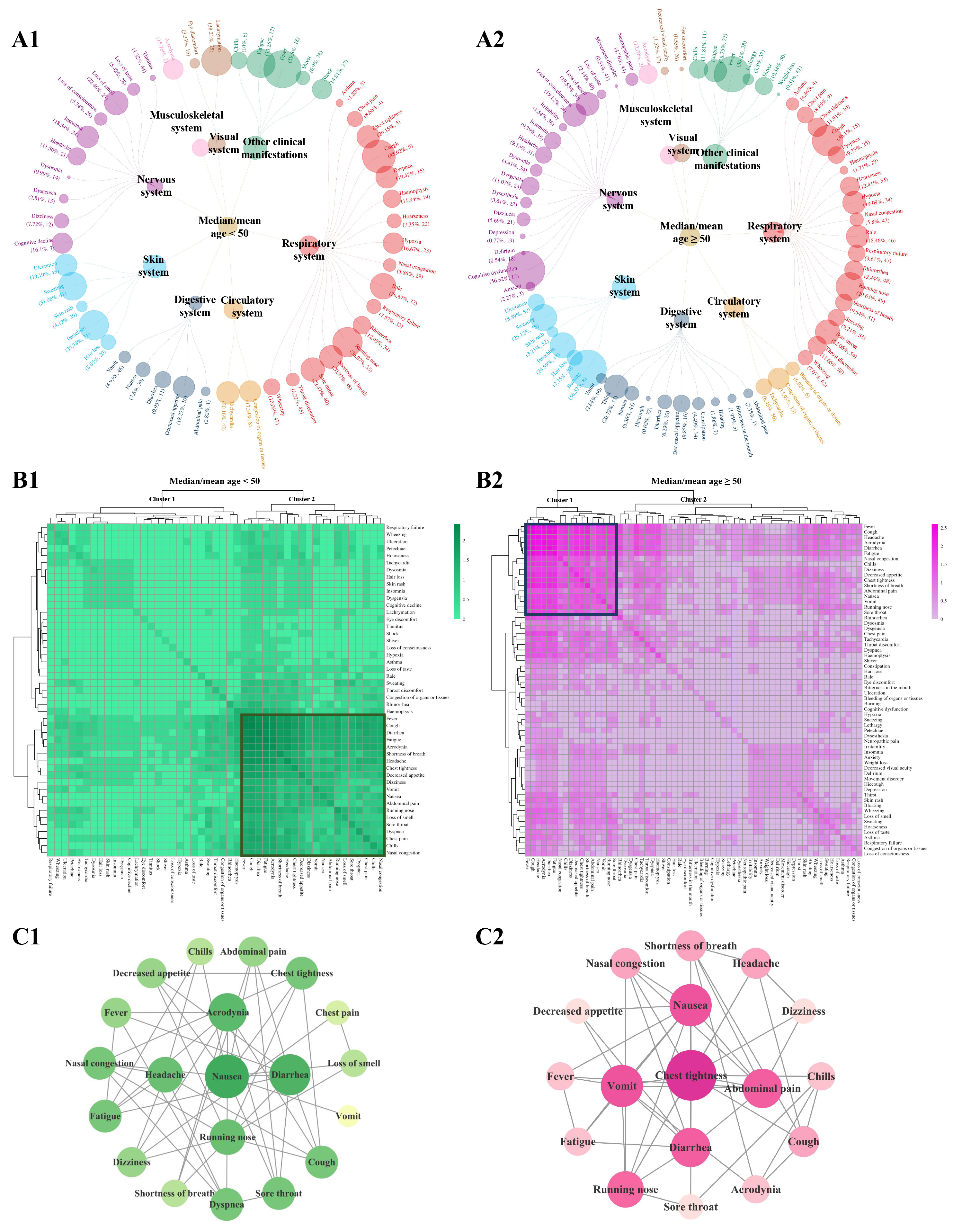

Supplement: Supplementary file 3 — Additional file 3. Fig. S3 Characteristics of COVID-19 symptom clusters in patients under and over 50 years of age in China. (A1) A total of 47 clinical symptoms were distributed in 7 disease systems and other clinical manifestations of COVID-19 in patients < 50 years; (B1) Cluster 2 was identified as the core symptom cluster in patients < 50 years, with varying shades of green indicating the frequency of co-occurring symptoms; (C1) Symptom correlation analysis showed nausea was the key symptom in patients < 50 years, and both the shade of green and the size of the circle represented the frequency of association between symptoms; (A2) A total of 62 clinical symptoms were distributed in 7 disease systems and other clinical manifestations of COVID-19 in patients ≥ 50 years; (B2) Cluster 1 was identified as the core symptom cluster in patients ≥ 50 years, with varying shades of purple indicating the frequency of co-occurring symptoms; (C2) Symptom correlation analysis showed chest tightness was the key symptom in patients ≥ 50 years, and both the shade of blue and the size of the circle represented the frequency of association between symptoms. [file 13020_2024_1043_MOESM3_ESM.jpg]

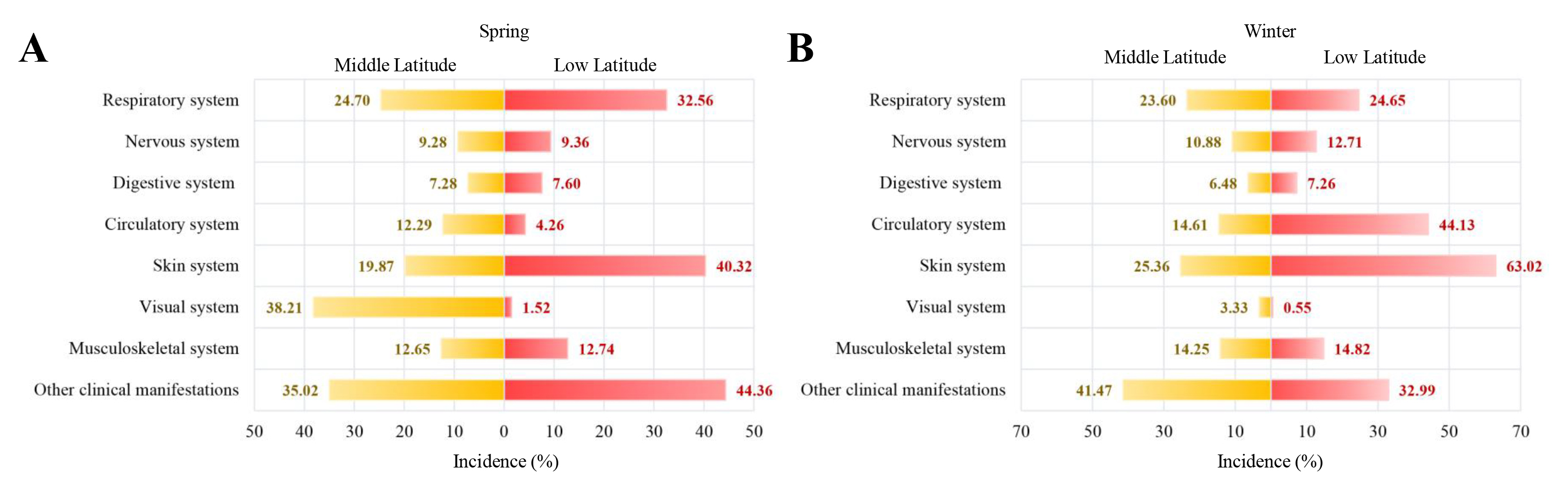

Supplement: Supplementary file 4 — Additional file 4. Fig. S4 Distribution characteristics of the disease system with clinical symptoms of COVID-19 in middle and low latitudes during (A) spring and (B) winter. [file 13020_2024_1043_MOESM4_ESM.jpg]

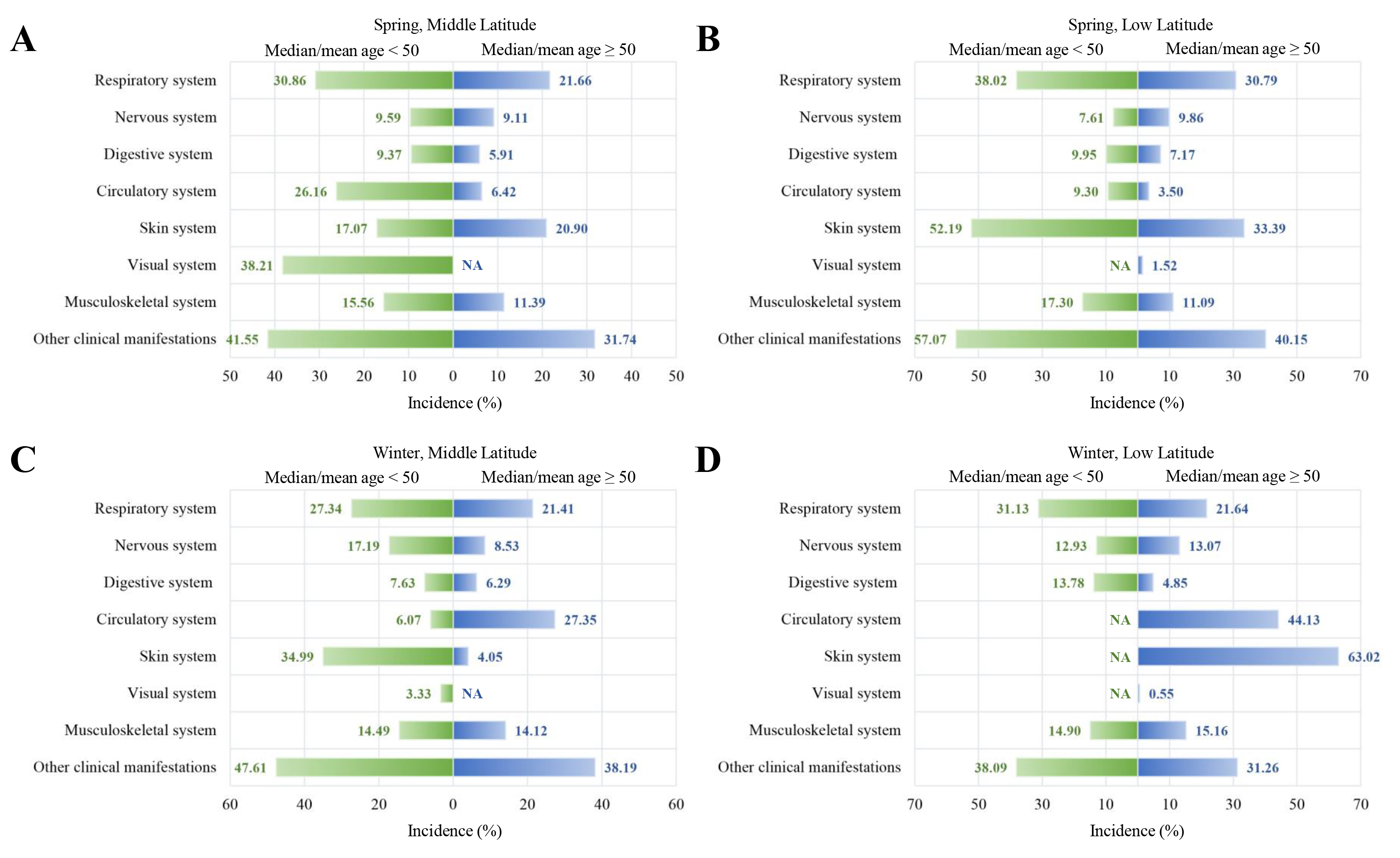

Supplement: Supplementary file 5 — Additional file 5. Fig. S5 Distribution characteristics of the disease system with clinical symptoms of COVID-19 in patients < 50 years old and ≥ 50 years old in middle (A)/low (B) latitudes during spring and (B) middle (C)/low (D) latitudes during winter. [file 13020_2024_1043_MOESM5_ESM.jpg]
