# Supplementary material for: Temporal, spatial and demographic distributions characteristics of COVID-19 symptom clusters from chinese medicine perspective: a systematic cross-sectional study in China from 2019 to 2023
Source: Chin Med. 2024 Dec 18;19:171. doi: 10.1186/s13020-024-01043-4 (PMC11654152; doi:10.1186/s13020-024-01043-4)
Supplement: Supplementary file 6 — Additional file 6. Table S1. [file 13020_2024_1043_MOESM6_ESM.docx]

**Table S1 Basic information of included 366 COVID-19 clinical trials.**

| **No.** | **Title** | **DOI** | **COVID-19 sample size** | **Start date** | **End date** | **Middle date** | **Season** | **Province of China** | **Latitude** | **Median/mean age** |
| --- | --- | --- | --- | --- | --- | --- | --- | --- | --- | --- |
| 1 | 15-Month Health Outcomes and the Related Risk Factors of Hospitalized COVID-19 Patients From Onset: A Cohort Study. | 10.3389/fmed.2022.854788 | 534 | 2020/2/20 | 2020/3/31 | 2020/3/11 | Spring | Shanghai | Middle latitude | ≥50 |
| 2 | 2019 novel coronavirus (COVID-19) pneumonia: CT manifestations and pattern of evolution in 110 patients in Jiangxi, China. | 10.1007/s00330-020-07201-0 | 110 | 2020/1/21 | 2020/2/5 | 2020/1/28 | Winter | Jiangxi | Low latitude | <50 |
| 3 | 2022年上海市某区定点医院151例新型冠状病毒肺炎病例临床特征分析 | 10.19428/j.cnki.sjpm.2022.22263 | 151 | 2022/4/13 | 2020/5/10 | 2021/4/26 | Spring | Shanghai | Middle latitude | ≥50 |
| 4 | 30例新型冠状病毒核酸复阳返院患者的病例特点 | 10.13406/j.cnki.cyxb.002558 | 90 | 2019/12/1 | 2020/3/11 | 2020/1/20 | Winter | Guangdong | Low latitude | ≥50 |
| 5 | 35例新型冠状病毒肺炎患者中医临床特征分析 | 10.16305/j.1007-1334.2020.04.095 | 35 | 2020/1/23 | 2020/2/14 | 2020/2/3 | Winter | Guangdong | Low latitude | <50 |
| 6 | A case series of COVID-19 patients with chronic hepatitis B virus infection | 10.1002/jmv.26201 | 28 | 2020/1/18 | 2020/2/26 | 2020/2/6 | Spring | Jiangsu | Middle latitude | <50 |
| 7 | A clinical pilot study on the safety and efficacy of aerosol inhalation treatment of IFN-κ plus TFF2 in patients with moderate COVID-19 | 10.1016/j.eclinm.2020.100478 | 33 | 2020/2/1 | 2020/4/6 | 2020/3/4 | Spring | Shanghai | Middle latitude | ≥50 |
| 8 | A clinical staging proposal of the disease course over time in non-severe patients with coronavirus disease 2019 | 10.1038/s41598-021-90111-y | 108 | 2020/2/10 | 2020/3/26 | 2020/3/3 | Spring | Fujian | Low latitude | ≥50 |
| 9 | A comparative study of the laboratory features of COVID-19 and other viral pneumonias in the recovery stage | 10.1002/jcla.23483 | 47 | 2020/1/31 | 2020/4/3 | 2020/3/2 | Spring | Shanxi-陕西 | Middle latitude | ≥50 |
| 10 | A comprehensive, longitudinal analysis of humoral responses specific to four recombinant antigens of SARS-CoV-2 in severe and non-severe COVID-19 patients | 10.1371/journal.ppat.1008796 | 26 | 2020/1/30 | 2020/2/25 | 2020/2/12 | Spring | Jiangsu | Middle latitude | <50 |
| 11 | A new rapid screening program based on risk scores for COVID-19 patients | 10.1007/s11739-020-02534-6 | 124 | 2020/2/8 | 2020/2/8 | 2020/2/8 | Spring | Hunan | Low latitude | <50 |
| 12 | A Novel Scoring System for Prediction of Disease Severity in COVID-19 | 10.3389/fcimb.2020.00318 | 80 | 2020/1/1 | 2020/2/29 | 2020/1/30 | Winter | Beijing | Middle latitude | ≥50 |
| 13 | A retrospective analysis from a single center for 60 COVID-19 patients with asymptomatic, mild to moderate, and severe conditions in Wuxi, China | 10.1097/MD.0000000000026748 | 60 | 2020/2/1 | 2020/3/31 | 2020/3/1 | Spring | Jiangsu | Middle latitude | <50 |
| 14 | A Retrospective Analysis of the Clinical and Epidemiological Characteristics of COVID-19 Patients in Henan Provincial People's Hospital, Zhengzhou, China. | 10.3389/fmed.2020.00286 | 29 | 2020/1/27 | 2020/4/1 | 2020/2/28 | Spring | Henan | Middle latitude | ≥50 |
| 15 | A retrospective observational study on maternal and neonatal outcomes of COVID-19: Does the mild SARS-CoV-2 infection affect the outcome? | 10.7717/peerj.16651 | 622 | 2022/11/1 | 2023/1/31 | 2022/12/16 | Winter | Guangdong | Low latitude | <50 |
| 16 | Absence of SARS-CoV-2 in semen of a COVID-19 patient cohort | 10.1111/andr.12848 | 23 | 2020/2/26 | 2020/4/2 | 2020/3/15 | Spring | Shandong | Middle latitude | <50 |
| 17 | Acupressure in the treatment of patients with mild infection of COVID-19 omicron variant: A prospectively observational study | 10.1097/MD.0000000000034610 | 800 | 2022/4/1 | 2022/5/1 | 2022/4/16 | Spring | Shanghai | Middle latitude | <50 |
| 18 | Acute Kidney Injury and Early Predictive Factors in COVID-19 Patients. | 10.3389/fmed.2021.604242 | 408 | 2020/1/1 | 2020/3/31 | 2020/2/15 | Spring | Guangdong | Low latitude | <50 |
| 19 | Adjusting intervention strategies for mental health of COVID-19 patients: A network analysis based on a survey in Omicron-infected patients | 10.3389/fpubh.2022.1038296 | 1239 | 2022/4/15 | 2022/4/25 | 2022/4/20 | Spring | Shanghai | Middle latitude | <50 |
| 20 | Adjuvant corticosteroid therapy for critically ill patients with COVID-19 | 10.1186/s13054-020-02964-w | 306 | 2020/1/25 | 2020/2/25 | 2020/2/9 | Spring | Jiangsu | Middle latitude | ≥50 |
| 21 | Air and surface contamination in non-health care settings among 641 environmental specimens of 39 COVID-19 cases | 10.1371/journal.pntd.0008570 | 39 | 2020/2/6 | 2020/4/10 | 2020/3/9 | Spring | Guangdong | Low latitude | <50 |
| 22 | Alterations in Fecal Fungal Microbiome of Patients With COVID-19 During Time of Hospitalization until Discharge | 10.1053/j.gastro.2020.06.048 | 30 | 2020/2/5 | 2020/5/12 | 2020/3/24 | Spring | Hong Kong | Low latitude | <50 |
| 23 | Alterations in Gut Microbiota of Patients With COVID-19 During Time of Hospitalization | 10.1053/j.gastro.2020.05.048 | 15 | 2020/2/5 | 2020/3/17 | 2020/2/25 | Spring | Hong Kong | Low latitude | ≥50 |
| 24 | An improved multivariate model that distinguishes COVID-19 from seasonal flu and other respiratory diseases. | 10.18632/aging.104132 | 19 | 2020/3/1 | 2020/4/30 | 2020/3/31 | Spring | Shanxi-山西 | Middle latitude | <50 |
| 25 | An open, prospective cohort study of VV116 in Chinese participants infected with SARS-CoV-2 omicron variants. | 10.1080/22221751.2022.2078230 | 136 | 2022/3/8 | 2022/3/24 | 2022/3/16 | Spring | Shanghai | Middle latitude | <50 |
| 26 | An open-label, randomized trial of the combination of IFN-κ plus TFF2 with standard care in the treatment of patients with moderate COVID-19 | 10.1016/j.eclinm.2020.100547 | 80 | 2020/3/23 | 2020/5/23 | 2020/4/22 | Spring | Shanghai | Middle latitude | <50 |
| 27 | Analysis of 394 COVID-19 cases infected with Omicron variant in Shenzhen: impact of underlying diseases to patient's symptoms | 10.1186/s40001-022-00927-1 | 786 | 2021/12/16 | 2022/3/24 | 2022/2/3 | Winter | Guangdong | Low latitude | <50 |
| 28 | Analysis of 4 imaging features in patients with COVID-19. | 10.1186/s12880-020-00484-1 | 43 | 2020/1/22 | 2020/2/26 | 2020/2/8 | Spring | Guangdong | Low latitude | ≥50 |
| 29 | Analysis of clinical features and early warning signs in patients with severe COVID-19: A retrospective cohort study | 10.1371/journal.pone.0235459 | 104 | 2019/12/1 | 2020/2/15 | 2020/1/8 | Winter | Henan | Middle latitude | <50 |
| 30 | Analysis of COVID-19 epidemic and clinical risk factors of patients under epidemiological Markov model | 10.1016/j.rinp.2021.103881 | 500 | 2020/1/1 | 2020/5/31 | 2020/3/16 | Spring | Shanxi-山西 | Middle latitude | <50 |
| 31 | Anemia is associated with severe illness in COVID-19: A retrospective cohort study | 10.1002/jmv.26444 | 222 | 2019/12/1 | 2020/3/20 | 2020/1/25 | Winter | Shanghai | Middle latitude | ≥50 |
| 32 | Antibody Responses and the Effects of Clinical Drugs in COVID-19 Patients | 10.3389/fimmu.2021.580989 | 36 | 2020/1/22 | 2020/3/7 | 2020/2/13 | Spring | Guangdong | Low latitude | ≥50 |
| 33 | Antiviral Efficacy and Safety of Molnupiravir Against Omicron Variant Infection: A Randomized Controlled Clinical Trial. | 10.3389/fphar.2022.939573 | 108 | 2022/3/3 | 2022/3/21 | 2022/3/12 | Spring | Guangdong | Low latitude | <50 |
| 34 | Artificial Intelligence Augmentation of Radiologist Performance in Distinguishing COVID-19 from Pneumonia of Other Origin at Chest CT | 10.1148/radiol.2020201491 | 521 | 2020/1/1 | 2020/4/28 | 2020/2/29 | Spring | Hunan | Low latitude | <50 |
| 35 | Assessment of Long-Term Effects on Pulmonary Functions Between Severe and Non-Severe Convalescent COVID-19 Patients: A Single-Center Study in China. | 10.2147/JIR.S371283 | 56 | 2020/2/11 | 2020/6/14 | 2020/4/13 | Spring | Beijing | Middle latitude | <50 |
| 36 | Assessment of SARS-CoV-2 Immunity in Convalescent Children and Adolescents | 10.3389/fimmu.2021.797919 | 31 | 2020/12/1 | 2021/3/31 | 2021/1/30 | Winter | Hong Kong | Low latitude | <50 |
| 37 | Association between detectable SARS-COV-2 RNA in anal swabs and disease severity in patients with coronavirus disease 2019 | 10.1002/jmv.26307 | 434 | 2020/1/20 | 2020/2/20 | 2020/2/4 | Spring | Guangdong | Low latitude | ≥50 |
| 38 | Association between Initial Chest CT or Clinical Features and Clinical Course in Patients with Coronavirus Disease 2019 Pneumonia | 10.3348/kjr.2020.0171 | 72 | 2020/1/16 | 2020/2/13 | 2020/1/30 | Winter | Shanxi-陕西 | Middle latitude | <50 |
| 39 | Association Between Respiratory Alkalosis and the Prognosis of COVID-19 Patients. | 10.3389/fmed.2021.564635 | 230 | 2020/1/17 | 2020/3/14 | 2020/2/14 | Spring | Hunan | Low latitude | ≥50 |
| 40 | Association between smoking and COVID-19 severity: A multicentre retrospective observational study | 10.1097/MD.0000000000029438 | 588 | 2020/1/10 | 2020/3/14 | 2020/2/11 | Spring | Jiangsu | Middle latitude | ≥50 |
| 41 | Association between the nasopharyngeal microbiome and metabolome in patients with COVID-19 | 10.1016/j.synbio.2021.06.002 | 9 | 2020/1/1 | 2020/2/28 | 2020/1/30 | Winter | Guangdong | Low latitude | <50 |
| 42 | Association of blood glucose level and prognosis of inpatients with coexistent diabetes and COVID-19 | 10.1007/s12020-021-02923-7 | 206 | 2020/2/4 | 2020/3/30 | 2020/3/2 | Spring | Jiangsu | Middle latitude | ≥50 |
| 43 | Association of cardiac injury with hypertension in hospitalized patients with COVID-19 in China. | 10.1038/s41598-021-01796-0 | 388 | 2020/1/16 | 2020/3/14 | 2020/2/14 | Spring | Hunan | Low latitude | <50 |
| 44 | Associations of clinical characteristics and treatment regimens with the duration of viral RNA shedding in patients with COVID-19 | 10.1016/j.ijid.2020.06.091 | 534 | 2020/1/20 | 2020/3/15 | 2020/2/16 | Spring | Guangdong | Low latitude | <50 |
| 45 | Associations of procalcitonin, C-reaction protein and neutrophil-to-lymphocyte ratio with mortality in hospitalized COVID-19 patients in China. | 10.1038/s41598-020-72164-7 | 76 | 2020/1/17 | 2020/2/15 | 2020/1/31 | Winter | Heilongjiang | Middle latitude | ≥50 |
| 46 | Asymptomatic COVID-19 Individuals Tend to Establish Relatively Balanced Innate and Adaptive Immune Responses. | 10.3390/pathogens10091105 | 41 | 2020/1/1 | 2020/6/28 | 2020/3/30 | Spring | Hebei | Middle latitude | <50 |
| 47 | Bacterial co-infections and antibiotic prescribing practice in adults with COVID-19: experience from a single hospital cluster. | 10.1177/2049936120978095 | 135 | 2020/1/8 | 2020/5/1 | 2020/3/5 | Spring | Hong Kong | Low latitude | <50 |
| 48 | Biologics targeting IL-17 and IL-23 maintain stability in patients with psoriasis during COVID-19 infection: a case-control study | 10.3389/fmed.2023.1280965 | 822 | 2022/12/1 | 2023/2/28 | 2023/1/14 | Winter | Shanghai | Middle latitude | ≥50 |
| 49 | Biomarkers Linked with Dynamic Changes of Renal Function in Asymptomatic and Mildly Symptomatic COVID-19 Patients. | 10.3390/jpm11050432 | 27 | 2020/2/4 | 2020/5/26 | 2020/3/31 | Spring | Taiwan | Low latitude | <50 |
| 50 | Blood Purification in Severe and Critical COVID-19 Patients: A Case Series of 5 Patients | 10.3389/fpubh.2021.741125 | 5 | 2020/1/31 | 2020/3/1 | 2020/2/15 | Spring | Guizhou | Low latitude | ≥50 |
| 51 | Cardiovascular sequalae in uncomplicated COVID-19 survivors | 10.1371/journal.pone.0246732 | 97 | 2020/4/1 | 2020/4/28 | 2020/4/14 | Spring | Hong Kong | Low latitude | <50 |
| 52 | Cautions on the laboratory indicators of COVID-19 patients on and during admission | 10.1002/jcla.23767 | 74 | 2020/1/1 | 2020/3/15 | 2020/2/7 | Spring | Hunan | Low latitude | <50 |
| 53 | Changes of gut microbiota under different nutritional methods in elderly patients with severe COVID-19 and their relationship with prognosis | 10.3389/fimmu.2023.1260112 | 142 | 2023/1/1 | 2023/2/28 | 2023/1/30 | Winter | Guangdong | Low latitude | ≥50 |
| 54 | Characteristic CT findings distinguishing 2019 novel coronavirus disease (COVID-19) from influenza pneumonia | 10.1007/s00330-020-06880-z | 13 | 2020/1/16 | 2020/2/25 | 2020/2/5 | Spring | Shanghai | Middle latitude | <50 |
| 55 | Characteristic of 523 COVID-19 in Henan Province and a Death Prediction Model | 10.3389/fpubh.2020.00475 | 523 | 2020/1/22 | 2020/2/20 | 2020/2/5 | Spring | Henan | Middle latitude | <50 |
| 56 | Characteristics and outcomes of patients with COVID-19 in Hainan, South China. | 10.1097/MD.0000000000024771 | 70 | 2020/1/1 | 2020/3/28 | 2020/2/13 | Spring | Hainan | Low latitude | ≥50 |
| 57 | Characteristics and prognostic factors of disease severity in patients with COVID-19: The Beijing experience | 10.1016/j.jaut.2020.102473 | 126 | 2019/12/1 | 2020/4/30 | 2020/2/14 | Spring | Beijing | Middle latitude | <50 |
| 58 | Characteristics and roles of severe acute respiratory syndrome coronavirus 2-specific antibodies in patients with different severities of coronavirus 19 | 10.1111/cei.13500 | 43 | 2020/2/1 | 2020/4/28 | 2020/3/15 | Spring | Guangdong | Low latitude | ≥50 |
| 59 | Characteristics of asymptomatic patients with SARS-CoV-2 infection in Jinan, China | 10.1016/j.micinf.2020.04.011 | 47 | 2020/1/23 | 2020/3/10 | 2020/2/15 | Spring | Shandong | Middle latitude | <50 |
| 60 | Characteristics of COVID-19 infection in Beijing | 10.1016/j.jinf.2020.02.018 | 262 | 2020/1/20 | 2020/2/10 | 2020/1/30 | Winter | Beijing | Middle latitude | <50 |
| 61 | Characteristics of respiratory virus infection during the outbreak of 2019 novel coronavirus in Beijing | 10.1016/j.ijid.2020.05.008 | 18 | 2020/1/19 | 2020/2/22 | 2020/2/5 | Spring | Beijing | Middle latitude | <50 |
| 62 | Chest computed tomography findings of coronavirus disease 2019 (COVID-19) pneumonia | 10.1007/s00330-020-06920-8 | 55 | 2020/1/9 | 2020/2/26 | 2020/2/2 | Winter | Henan | Middle latitude | <50 |
| 63 | Chest CT-based differential diagnosis of 28 patients with suspected corona virus disease 2019 (COVID-19) | 10.1259/bjr.20200243 | 12 | 2020/1/22 | 2020/2/6 | 2020/1/29 | Winter | Guangdong | Low latitude | <50 |
| 64 | Clinical and CT characteristics which indicate timely radiological reexamination in patients with COVID-19: A retrospective study in Beijing, China | 10.1016/j.jrid.2020.05.003 | 73 | 2020/1/21 | 2020/2/24 | 2020/2/7 | Spring | Beijing | Middle latitude | ≥50 |
| 65 | Clinical and CT features of the COVID-19 infection: comparison among four different age groups | 10.1007/s41999-020-00356-5 | 97 | 2020/1/17 | 2020/2/21 | 2020/2/3 | Winter | Guangdong | Low latitude | <50 |
| 66 | Clinical and imaging features of pediatric COVID-19. | 10.1186/s13052-020-00917-1 | 41 | 2020/1/1 | 2020/2/28 | 2020/1/30 | Winter | Hebei | Middle latitude | ≥50 |
| 67 | Clinical and imaging findings of discharged patients with SARS-CoV-2 positive anal swab samples: a descriptive study. | 10.1186/s12879-020-05363-2 | 84 | 2020/1/20 | 2020/2/22 | 2020/2/5 | Spring | Hunan | Low latitude | <50 |
| 68 | Clinical and psychological status analysis of children and parents infected with familial aggregation omicron variant in Shanghai in parent-child ward | 10.1016/j.heliyon.2022.e12151 | 455 | 2022/4/8 | 2022/5/31 | 2022/5/4 | Spring | Shanghai | Middle latitude | <50 |
| 69 | Clinical and radiological changes of hospitalised patients with COVID-19 pneumonia from disease onset to acute exacerbation: a multicentre paired cohort study. | 10.1007/s00330-020-06916-4 | 48 | 2019/12/1 | 2020/2/16 | 2020/1/8 | Winter | Hunan | Low latitude | ≥50 |
| 70 | Clinical and virological course of patients with coronavirus disease 2019 in Jiangsu province, China: a retrospective, multi-center cohort study | 10.1186/s12985-021-01615-y | 328 | 2020/1/18 | 2020/2/29 | 2020/2/8 | Spring | Jiangsu | Middle latitude | <50 |
| 71 | Clinical Characteristics and Abnormal Parameters Evolution in Patients With Novel Coronavirus Infection: A Case Series of 272 Cases in Guangzhou. | 10.1017/dmp.2021.149 | 272 | 2020/1/22 | 2020/2/15 | 2020/2/3 | Winter | Guangdong | Low latitude | <50 |
| 72 | Clinical characteristics and changes of chest CT features in 307 patients with common COVID-19 pneumonia infected SARS-CoV-2 A multicenter study in Jiangsu, China | 10.1016/j.ijid.2020.05.006 | 307 | 2020/1/10 | 2020/3/3 | 2020/2/5 | Spring | Jiangsu | Middle latitude | <50 |
| 73 | Clinical characteristics and chest CT imaging features of critically ill COVID-19 patients | 10.1007/s00330-020-06955-x | 60 | 2020/1/9 | 2020/2/19 | 2020/1/29 | Winter | Hunan | Low latitude | ≥50 |
| 74 | Clinical Characteristics and Eosinophils in Young SARS-CoV-2-Positive Chinese Travelers Returning to Shanghai. | 10.3389/fpubh.2020.00368 | 36 | 2020/3/1 | 2020/3/23 | 2020/3/12 | Spring | Shanghai | Middle latitude | <50 |
| 75 | Clinical characteristics and laboratory indicator analysis of 67 COVID-19 pneumonia patients in Suzhou, China. | 10.1186/s12879-020-05468-8 | 67 | 2020/1/1 | 2020/2/8 | 2020/1/20 | Winter | Jiangsu | Middle latitude | <50 |
| 76 | Clinical characteristics and outcomes of 48 patients hospitalized for COVID-19 infection in Wuxi A retrospective cohort study | 10.1097/MD.0000000000023991 | 48 | 2020/1/23 | 2020/3/8 | 2020/2/14 | Spring | Jiangsu | Middle latitude | <50 |
| 77 | Clinical characteristics and outcomes of critically ill patients with acute COVID-19 with Epstein-Barr virus reactivation | 10.1186/s12879-021-06638-y | 128 | 2020/1/31 | 2020/3/27 | 2020/2/28 | Spring | Shanghai | Middle latitude | ≥50 |
| 78 | Clinical Characteristics and Predictors of Disease Progression in Severe Patients with COVID-19 Infection in Jiangsu Province, China: A Descriptive Study. | 10.1016/j.amjms.2020.05.038 | 60 | 2020/1/24 | 2020/4/20 | 2020/3/7 | Spring | Jiangsu | Middle latitude | ≥50 |
| 79 | Clinical Characteristics and Prognosis of 218 Patients With COVID-19: A Retrospective Study Based on Clinical Classification. | 10.3389/fmed.2020.00485 | 218 | 2020/1/21 | 2020/6/27 | 2020/4/9 | Spring | Hunan | Low latitude | <50 |
| 80 | Clinical Characteristics and Reasons for Differences in Duration From Symptom Onset to Release From Quarantine Among Patients With COVID-19 in Liaocheng, China. | 10.3389/fmed.2020.00210 | 37 | 2019/12/1 | 2020/3/1 | 2020/1/15 | Winter | Shandong | Middle latitude | <50 |
| 81 | Clinical characteristics and risk factors for severity of COVID-19 outside Wuhan: a double-center retrospective cohort study of 213 cases in Hunan, China | 10.1177/1753466620963035 | 213 | 2020/1/24 | 2020/2/16 | 2020/2/4 | Spring | Hunan | Low latitude | <50 |
| 82 | Clinical characteristics and risk factors of mild-to-moderate COVID-19 patients with false-negative SARS-CoV-2 nucleic acid | 10.1002/jmv.26242 | 50 | 2020/2/3 | 2020/3/7 | 2020/2/19 | Spring | Guangdong | Low latitude | <50 |
| 83 | Clinical characteristics and risk factors of patients with severe COVID-19 in Jiangsu province, China: a retrospective multicentre cohort study | 10.1186/s12879-020-05314-x | 625 | 2020/1/10 | 2020/3/15 | 2020/2/11 | Spring | Jiangsu | Middle latitude | <50 |
| 84 | Clinical Characteristics in Patients with Redetected Positive RNA Test After Recovery from Foreign-Imported COVID-19 Cases in Xi'an, China. | 10.2147/IDR.S371088 | 27 | 2020/7/28 | 2021/12/31 | 2021/4/14 | Spring | Shanxi-陕西 | Middle latitude | <50 |
| 85 | Clinical characteristics of 310 SARS-CoV-2 Omicron variant patients and comparison with Delta and Beta variant patients in China. | 10.1016/j.virs.2022.07.014 | 310 | 2022/1/8 | 2020/1/18 | 2021/1/12 | Winter | Tianjin | Middle latitude | <50 |
| 86 | Clinical characteristics of 41 patients with pneumonia due to 2019 novel coronavirus disease (COVID-19) in Jilin, China. | 10.1186/s12879-020-05677-1 | 41 | 2020/1/1 | 2020/2/25 | 2020/1/28 | Winter | Jilin | Middle latitude | <50 |
| 87 | Clinical characteristics of COVID-19 in children compared with adults in Shandong Province, China | 10.1007/s15010-020-01427-2 | 67 | 2020/1/23 | 2020/2/15 | 2020/2/3 | Winter | Shandong | Middle latitude | <50 |
| 88 | Clinical Characteristics of COVID-19 Patients Infected by the Omicron Variant of SARS-CoV-2. | 10.3389/fmed.2022.912367 | 338 | 2020/2/1 | 2022/3/28 | 2021/2/28 | Spring | Jiangsu | Middle latitude | <50 |
| 89 | Clinical Characteristics of COVID-19 Patients With Digestive Symptoms in Hubei, China: A Descriptive, Cross-Sectional, Multicenter Study | 10.14309/ajg.0000000000000620 | 103 | 2020/1/18 | 2020/3/18 | 2020/2/17 | Spring | Beijing | Middle latitude | ≥50 |
| 90 | Clinical characteristics of foreign-imported COVID-19 cases in Shanghai, China. | 10.1080/22221751.2020.1766383 | 58 | 2020/3/5 | 2020/3/22 | 2020/3/13 | Spring | Shanghai | Middle latitude | <50 |
| 91 | Clinical characteristics of imported and second-generation coronavirus disease 2019 (COVID-19) cases in Shaanxi outside Wuhan, China: a multicentre retrospective study | 10.1017/S0950268820002332 | 134 | 2020/1/23 | 2020/3/7 | 2020/2/14 | Spring | Shanxi-陕西 | Middle latitude | <50 |
| 92 | Clinical Characteristics of Imported Cases of Coronavirus Disease 2019 (COVID-19) in Jiangsu Province: A Multicenter Descriptive Study | 10.1093/cid/ciaa199 | 80 | 2020/1/22 | 2020/2/14 | 2020/2/2 | Winter | Jiangsu | Middle latitude | <50 |
| 93 | Clinical Characteristics of Omicron SARS-CoV-2 Variant Infection After Non-mRNA-Based Vaccination in China. | 10.3389/fmicb.2022.901826 | 380 | 2022/1/8 | 2022/1/29 | 2022/1/18 | Winter | Henan | Middle latitude | <50 |
| 94 | Clinical characteristics of patients diagnosed with COVID-19 in Beijing | 10.1016/j.bsheal.2020.05.003 | 45 | 2020/1/28 | 2020/2/23 | 2020/2/10 | Spring | Beijing | Middle latitude | ≥50 |
| 95 | Clinical characteristics of pediatric cases infected with the SARS-CoV-2 Omicron variant in a tertiary children’s medical center in Shanghai, China | 10.1007/s12519-022-00621-6 | 676 | 2020/3/28 | 2022/4/30 | 2021/4/13 | Spring | Shanghai | Middle latitude | <50 |
| 96 | Clinical characteristics of pediatric inpatients infected with the SARS-CoV-2 Omicron variant- a retrospective observational cohort study | 10.21037/tp-23-292 | 98 | 2020/11/19 | 2022/11/30 | 2021/11/24 | Winter | Tianjin | Middle latitude | <50 |
| 97 | Clinical characteristics of re-positive discharged COVID-19 pneumonia patients in Wuhan, China | 10.1038/s41598-020-74284-6 | 267 | 2020/1/1 | 2020/2/15 | 2020/1/23 | Winter | Hunan | Low latitude | ≥50 |
| 98 | Clinical Characteristics of Severe COVID-19 Patients During Omicron Epidemic and a Nomogram Model Integrating Cell-Free DNA for Predicting Mortality: A Retrospective Analysis | 10.2147/IDR.S430101 | 846 | 2022/12/17 | 2023/1/27 | 2023/1/6 | Winter | Jiangsu | Middle latitude | <50 |
| 99 | Clinical Factors Associated with Progression and Prolonged Viral Shedding in COVID-19 Patients: A Multicenter Study. | 10.14336/AD.2020.0630 | 564 | 2020/1/17 | 2020/2/28 | 2020/2/7 | Spring | Hunan | Low latitude | <50 |
| 100 | Clinical features and development of sepsis in patients infected with SARS-CoV-2: a retrospective analysis of 150 cases outside Wuhan, China | 10.1007/s00134-020-06084-5 | 150 | 2020/1/11 | 2020/3/11 | 2020/2/10 | Spring | Jiangsu | Middle latitude | ≥50 |
| 101 | Clinical features and dynamics of viral load in imported and non-imported patients with COVID-19 | 10.1016/j.ijid.2020.03.022 | 51 | 2020/1/23 | 2020/2/18 | 2020/2/5 | Spring | Jiangsu | Middle latitude | <50 |
| 102 | Clinical features and prognosis of COVID-19 patients with metabolic syndrome: A multicenter, retrospective study | 10.1016/j.medcli.2021.05.014 | 233 | 2020/1/18 | 2020/2/26 | 2020/2/6 | Spring | Jiangsu | Middle latitude | <50 |
| 103 | Clinical features and risk factors for ICU admission in COVID-19 patients with cardiovascular diseases. | 10.14336/AD.2020.0622 | 288 | 2020/1/15 | 2020/3/10 | 2020/2/11 | Spring | Guangdong | Low latitude | <50 |
| 104 | Clinical features and risk factors for severe-critically ill COVID-19 adult patients in Jiangsu, China A multiple-centered, retrospective study | 10.1097/MD.0000000000024332 | 583 | 2019/11/1 | 2020/3/13 | 2020/1/6 | Winter | Jiangsu | Middle latitude | ≥50 |
| 105 | Clinical features of patients with coronavirus disease 2019 from a designated hospital in Beijing, China | 10.1002/jmv.25966 | 165 | 2020/1/20 | 2020/2/15 | 2020/2/2 | Winter | Beijing | Middle latitude | <50 |
| 106 | Clinical Findings of COVID-19 Patients Admitted to Intensive Care Units in Guangdong Province, China: A Multicenter, Retrospective, Observational Study. | 10.3389/fmed.2020.576457 | 45 | 2020/1/14 | 2020/2/20 | 2020/2/1 | Winter | Guangdong | Low latitude | ≥50 |
| 107 | Clinical imaging characteristics of inpatients with coronavirus disease-2019 in Heilongjiang Province, China: a retrospective study | 10.18632/aging.103633 | 59 | 2019/12/1 | 2020/5/4 | 2020/2/16 | Spring | Heilongjiang | Middle latitude | ≥50 |
| 108 | Clinical impact of monocyte distribution width and neutrophil-to-lymphocyte ratio for distinguishing COVID-19 and influenza from other upper respiratory tract infections: A pilot study | 10.1371/journal.pone.0241262 | 9 | 2020/2/19 | 2020/4/30 | 2020/3/25 | Spring | Taiwan | Low latitude | <50 |
| 109 | Clinical Observation and evaluation of Jinhua Qinggan Granules in the Treatment of Novel Coronavirus Pneumonia | 10.1016/j.jep.2022.115763 | 36 | 2020/1/24 | 2020/2/17 | 2020/2/5 | Spring | Beijing | Middle latitude | ≥50 |
| 110 | Clinical Potential of UTE-MRI for Assessing COVID-19: Patient- and Lesion-Based Comparative Analysis | 10.1002/jmri.27208 | 23 | 2020/2/1 | 2020/4/28 | 2020/3/15 | Spring | Shanghai | Middle latitude | <50 |
| 111 | Clinical Progression and Outcome of Hospitalized Patients Infected with SARS-CoV-2 Omicron Variant in Shanghai, China. | 10.3390/vaccines10091409 | 226 | 2022/4/6 | 2022/5/11 | 2022/4/23 | Spring | Shanghai | Middle latitude | ≥50 |
| 112 | Clinical progression of patients with COVID-19 in Shanghai, China | 10.1016/j.jinf.2020.03.004 | 249 | 2020/1/20 | 2020/2/6 | 2020/1/28 | Winter | Shanghai | Middle latitude | ≥50 |
| 113 | Clinical Time Features and Chest Imaging of 85 Patients With COVID-19 in Zhuhai, China. | 10.3389/fmed.2020.00209 | 85 | 2020/1/17 | 2020/2/11 | 2020/1/29 | Winter | Guangdong | Low latitude | <50 |
| 114 | Clinical Utility of a Nomogram for Predicting 30-Days Poor Outcome in Hospitalized Patients With COVID-19: Multicenter External Validation and Decision Curve Analysis. | 10.3389/fmed.2020.590460 | 233 | 2020/1/3 | 2020/3/20 | 2020/2/10 | Spring | Guangdong | Low latitude | ≥50 |
| 115 | Clinical, laboratory, and radiological features indicative of novel coronavirus disease (COVID-19) in emergency departments: a multicenter case-control study in Hong Kong. | 10.1002/emp2.12183 | 37 | 2020/1/20 | 2020/2/29 | 2020/2/9 | Spring | Hong Kong | Low latitude | ≥50 |
| 116 | Co-Infection with Common Respiratory Pathogens and SARS-CoV-2 in Patients with COVID-19 Pneumonia and Laboratory Biochemistry Findings: A Retrospective Cross-Sectional Study of 78 Patients from a Single Center in China | 10.12659/MSM.929783 | 78 | 2020/1/28 | 2020/3/15 | 2020/2/20 | Spring | Hunan | Low latitude | <50 |
| 117 | Coinfection with SARS-CoV-2 and other respiratory pathogens in patients with COVID-19 in Guangzhou, China. | 10.1002/jmv.26073 | 18 | 2020/1/1 | 2020/3/1 | 2020/1/31 | Winter | Guangdong | Low latitude | <50 |
| 118 | Combined Interventions for Severe Novel Coronavirus Disease (COVID-19): Experience from 350 Patients. | 10.2147/IDR.S279255 | 350 | 2020/1/21 | 2020/2/19 | 2020/2/4 | Spring | Hunan | Low latitude | <50 |
| 119 | Comparative Analysis of Early-Stage Clinical Features Between COVID-19 and Influenza A H1N1 Virus Pneumonia | 10.3389/fpubh.2020.00206 | 15 | 2020/1/22 | 2020/2/20 | 2020/2/5 | Spring | Shanghai | Middle latitude | ≥50 |
| 120 | Comparative Study of Acute Lung Injury in COVID-19 and Non-COVID-19 Patients. | 10.3389/fmed.2021.666629 | 90 | 2020/1/1 | 2020/4/28 | 2020/2/29 | Spring | Jiangsu | Middle latitude | ≥50 |
| 121 | Comparative study on the clinical characteristics of local cases of COVID-19 and imported cases from abroad A retrospective cohort study | 10.1097/MD.0000000000026933 | 160 | 2020/2/29 | 2020/3/27 | 2020/3/13 | Spring | Beijing | Middle latitude | <50 |
| 122 | Comparative therapeutic efficacy of interferon alfa-2b and combination lopinavir/ritonavir plus interferon alfa-2b against SARS-CoV-2. | 10.1186/s12879-021-06595-6 | 123 | 2020/1/13 | 2020/4/23 | 2020/3/3 | Spring | Beijing | Middle latitude | <50 |
| 123 | Compare the epidemiological and clinical features of imported and local COVID-19 cases in Hainan, China. | 10.1186/s40249-020-00755-7 | 91 | 2020/1/20 | 2020/2/19 | 2020/2/4 | Spring | Hainan | Low latitude | ≥50 |
| 124 | Comparison of adverse effects of anti-tumor therapy for breast cancer shortly after COVID-19 diagnosis vs. the control period | 10.3389/fonc.2023.1203119 | 218 | 2022/12/20 | 2023/1/20 | 2023/1/4 | Winter | Beijing | Middle latitude | ≥50 |
| 125 | Comparison of Clinical and Epidemiological Characteristics of Asymptomatic and Symptomatic SARS-CoV-2 Infection in Children. | 10.1007/s12250-020-00312-4 | 98 | 2020/1/19 | 2020/4/30 | 2020/3/10 | Spring | Shanghai | Middle latitude | <50 |
| 126 | Comparison of Clinical Characteristics and Outcomes of Pediatric and Adult Patients with Coronavirus Disease 2019 in Shenzhen, China. | 10.3967/bes2020.124 | 333 | 2020/1/11 | 2020/2/10 | 2020/1/26 | Winter | Guangdong | Low latitude | <50 |
| 127 | Comparison of Clinical Characteristics and Peripheral Blood Tests of COVID-19 and Influenza B Patients. | 10.4269/ajtmh.22-0620 | 602 | 2022/1/1 | 2022/6/30 | 2022/4/1 | Spring | Shanghai | Middle latitude | ≥50 |
| 128 | Comparison of clinical characteristics between patients with coronavirus disease 2019 (COVID-19) who retested RT-PCR positive versus negative: a retrospective study of data from Nanjing. | 10.21037/jtd.2020.04.17 | 10 | 2020/1/20 | 2020/2/16 | 2020/2/2 | Winter | Jiangsu | Middle latitude | <50 |
| 129 | Comparison of clinical, laboratory, and radiological characteristics between SARS-CoV-2 infection and community-acquired pneumonia caused by influenza virus A cross-sectional retrospective study | 10.1097/MD.0000000000023064 | 35 | 2020/1/1 | 2020/3/4 | 2020/2/1 | Winter | Fujian | Low latitude | ≥50 |
| 130 | Comparison of patients hospitalized with COVID-19, H7N9 and H1N1. | 10.1186/s40249-020-00781-5 | 83 | 2020/1/17 | 2020/3/20 | 2020/2/17 | Spring | Guangdong | Low latitude | ≥50 |
| 131 | Comparison of qualitative and quantitative analyses of COVID-19 clinical samples | 10.1016/j.cca.2020.08.033 | 30 | 2020/2/1 | 2020/4/28 | 2020/3/15 | Spring | Beijing | Middle latitude | ≥50 |
| 132 | Comparison of the clinical characteristics of patients with novel coronavirus pneumonia between China and overseas | 10.1002/jcla.23811 | 90 | 2020/1/23 | 2020/5/1 | 2020/3/12 | Spring | Fujian | Low latitude | <50 |
| 133 | Comparison of the duration of viral RNA shedding and anti-SARS-CoV-2 spike IgG and IgM antibody titers in COVID-19 patients who were vaccinated with inactivated vaccines or not: a retrospective study | 10.1186/s12879-022-07808-2 | 147 | 2020/8/7 | 2021/8/20 | 2021/2/12 | Spring | Jiangsu | Middle latitude | ≥50 |
| 134 | Coronavirus disease 2019 (COVID-19) associated coagulopathy and its impact on outcomes in Shenzhen, China: A retrospective cohort study | 10.1016/j.thromres.2020.07.015 | 117 | 2020/1/14 | 2020/2/11 | 2020/1/28 | Winter | Guangdong | Low latitude | ≥50 |
| 135 | Correlation between chest CT severity score and laboratory indicators in patients with Coronavirus disease 2019 (COVID-19) | 10.1111/ijcp.14907 | 56 | 2020/1/24 | 2020/3/6 | 2020/2/14 | Spring | Fujian | Low latitude | <50 |
| 136 | Correlation between early features and prognosis of symptomatic COVID-19 discharged patients in Hunan, China | 10.1038/s41598-021-83654-7 | 172 | 2020/1/10 | 2020/2/28 | 2020/2/3 | Winter | Hunan | Low latitude | <50 |
| 137 | Corticosteroid prevents COVID-19 progression within its therapeutic window: a multicentre, proof-of-concept, observational study | 10.1080/22221751.2020.1807885 | 68 | 2020/1/20 | 2020/2/13 | 2020/2/1 | Winter | Shanghai | Middle latitude | ≥50 |
| 138 | Corticosteroid therapy is associated with the delay of SARS-CoV-2 clearance in COVID-19 patients | 10.1016/j.ejphar.2020.173556 | 309 | 2020/1/18 | 2020/2/26 | 2020/2/6 | Spring | Jiangsu | Middle latitude | <50 |
| 139 | Corticosteroid Use in the Treatment of COVID-19: A Multicenter Retrospective Study in Hunan, China. | 10.3389/fphar.2020.01198 | 982 | 2020/1/23 | 2020/3/8 | 2020/2/14 | Spring | Hunan | Low latitude | <50 |
| 140 | COVID-19 Cases from the First Local Outbreak of the SARS-CoV-2 B.1.1.7 Variant in China May Present More Serious Clinical Features: A Prospective, Comparative Cohort Study | 10.1128/Spectrum.00273-21 | 148 | 2020/12/25 | 2021/1/17 | 2021/1/5 | Winter | Beijing | Middle latitude | <50 |
| 141 | COVID-19 in Children with Cancer and Continuation of Cancer-Directed Therapy During the Infection. | 10.1007/s12098-021-03894-3 | 122 | 2020/3/1 | 2020/2/14 | 2020/2/22 | Spring | Henan | Middle latitude | <50 |
| 142 | Covid-19 vaccine effectiveness during Omicron BA.2 pandemic in Shanghai: A cross-sectional study based on EMR. | 10.1097/MD.0000000000031763 | 1194 | 2022/3/23 | 2022/4/1 | 2022/3/27 | Spring | Shanghai | Middle latitude | <50 |
| 143 | COVID-19 Viral Load in the Severity of and Recovery From Olfactory and Gustatory Dysfunction. | 10.1002/lary.29056 | 83 | 2020/2/8 | 2020/4/15 | 2020/3/12 | Spring | Hong Kong | Low latitude | <50 |
| 144 | COVID-19患者康复早期临床特征、肺功能及影像学随访 | 10.3969/j.issn.2095-5227.2021.04.002 | 38 | 2020/1/20 | 2020/4/2 | 2020/2/25 | Spring | Beijing | Middle latitude | <50 |
| 145 | COVID-19患者胸部CT表现及动态演变 | 10.12122/j.issn.1673-4254.2020.03.05 | 6 | 2020/1/27 | 2020/2/26 | 2020/2/11 | Spring | Shanxi-陕西 | Middle latitude | <50 |
| 146 | CT differential diagnosis of COVID-19 and non-COVID-19 in symptomatic suspects: a practical scoring method. | 10.1186/s12890-020-1170-6 | 30 | 2020/1/10 | 2020/2/28 | 2020/2/3 | Winter | Guangdong | Low latitude | ≥50 |
| 147 | CT features of COVID-19 patients with two consecutive negative RT-PCR tests after treatment | 10.1038/s41598-020-68509-x | 46 | 2020/2/16 | 2020/3/8 | 2020/2/26 | Spring | Guangxi | Low latitude | <50 |
| 148 | CT findings of patients infected with SARS-CoV-2. | 10.1186/s12880-020-00471-6 | 67 | 2020/1/23 | 2020/2/10 | 2020/2/1 | Winter | Shanghai | Middle latitude | <50 |
| 149 | CT imaging features of 34 patients infected with COVID-19 | 10.1016/j.clinimag.2020.05.016 | 34 | 2020/1/24 | 2020/3/5 | 2020/2/13 | Spring | Shandong | Middle latitude | <50 |
| 150 | CT Scans of Patients with 2019 Novel Coronavirus (COVID-19) Pneumonia | 10.7150/thno.45016 | 118 | 2020/1/16 | 2020/2/4 | 2020/1/25 | Winter | Hunan | Low latitude | <50 |
| 151 | Decline in neutralising antibody responses, but sustained T-cell immunity, in COVID-19 patients at 7 months post-infection. | 10.1002/cti2.1319 | 27 | 2020/1/1 | 2020/2/28 | 2020/1/30 | Winter | Shanghai | Middle latitude | ≥50 |
| 152 | Delayed discharge is associated with higher complement C3 levels and a longer nucleic acid-negative conversion time in patients with COVID-19. | 10.1038/s41598-021-81010-3 | 47 | 2020/1/21 | 2020/3/6 | 2020/2/12 | Spring | Fujian | Low latitude | <50 |
| 153 | Determinants of Increased Fibrinogen in COVID-19 Patients With and Without Diabetes and Impaired Fasting Glucose | 10.1177/1076029621996445 | 172 | 2020/2/9 | 2020/2/28 | 2020/2/18 | Spring | Beijing | Middle latitude | ≥50 |
| 154 | Determinants of post-COVID-19 symptoms among adults aged 55 or above with chronic conditions in primary care: data from a prospective cohort in Hong Kong. | 10.3389/fpubh.2023.1138147 | 414 | 2019/11/1 | 2022/5/28 | 2021/2/12 | Spring | Hong Kong | Low latitude | <50 |
| 155 | Determination of risk factors for predicting the onset of symptoms in asymptomatic COVID-19 infected patients | 10.7150/ijms.47576 | 140 | 2020/1/28 | 2020/2/18 | 2020/2/7 | Spring | Hunan | Low latitude | <50 |
| 156 | Development and Validation of a Deep Learning-Based Model Using Computed Tomography Imaging for Predicting Disease Severity of Coronavirus Disease 2019. | 10.3389/fbioe.2020.00898 | 408 | 2020/1/1 | 2020/3/18 | 2020/2/8 | Spring | Jiangxi | Low latitude | <50 |
| 157 | Development and Validation of a Prognostic Nomogram Based on Clinical and CT Features for Adverse Outcome Prediction in Patients with COVID-19 | 10.3348/kjr.2020.0485 | 238 | 2020/1/21 | 2020/3/3 | 2020/2/11 | Spring | Shanghai | Middle latitude | <50 |
| 158 | Development and validation of a simple tool composed of items on dyspnea, respiration rates, and C-reactive protein for pneumonia prediction among acute febrile respiratory illness patients in primary care settings | 10.1186/s12916-022-02552-5 | 727 | 2020/3/1 | 2021/12/31 | 2021/1/30 | Winter | Shanghai | Middle latitude | <50 |
| 159 | Development and validation of risk prediction models for COVID-19 positivity in a hospital setting | 10.1016/j.ijid.2020.09.022 | 212 | 2020/1/1 | 2020/4/1 | 2020/2/15 | Spring | Hong Kong | Low latitude | <50 |
| 160 | Development of a data-driven COVID-19 prognostication tool to inform triage and step-down care for hospitalised patients in Hong Kong: a population-based cohort study. | 10.1186/s12911-020-01338-0 | 1037 | 2019/12/1 | 2020/4/30 | 2020/2/14 | Spring | Hong Kong | Low latitude | <50 |
| 161 | Development of a prediction score (ThyroCOVID) for identifying abnormal thyroid function in COVID-19 patients | 10.1007/s40618-022-01854-y | 546 | 2020/7/21 | 2021/5/20 | 2020/12/19 | Winter | Hong Kong | Low latitude | ≥50 |
| 162 | Diagnostic performance between CT and initial real-time RT-PCR for clinically suspected 2019 coronavirus disease (COVID-19) patients outside Wuhan, China | 10.1016/j.rmed.2020.105980 | 34 | 2020/1/10 | 2020/2/28 | 2020/2/3 | Winter | Guangdong | Low latitude | ≥50 |
| 163 | Differences in Clinical Features and Laboratory Results between Adults and Children with SARS-CoV-2 Infection | 10.1155/2020/6342598 | 52 | 2020/2/1 | 2020/3/20 | 2020/2/25 | Spring | Guangdong | Low latitude | <50 |
| 164 | Differential Diagnosis of COVID-19 Pneumonia From Influenza A (H1N1) Pneumonia Using a Model Based on Clinicoradiologic Features. | 10.3389/fmed.2021.651556 | 291 | 2020/1/20 | 2020/2/13 | 2020/2/1 | Winter | Shanghai | Middle latitude | ≥50 |
| 165 | Differentiation of COVID-19 from seasonal influenza: A multicenter comparative study | 10.1002/jmv.26469 | 211 | 2020/1/1 | 2020/4/28 | 2020/2/29 | Spring | Jiangsu | Middle latitude | ≥50 |
| 166 | Digital PCR is a sensitive new technique for SARS-CoV-2 detection in clinical applications | 10.1016/j.cca.2020.10.032 | 10 | 2020/1/1 | 2020/4/28 | 2020/2/29 | Spring | Jilin | Middle latitude | ≥50 |
| 167 | Discriminant models for the prediction of postponed viral shedding time and disease progression in COVID-19. | 10.1186/s12879-022-07338-x | 125 | 2020/1/22 | 2020/3/22 | 2020/2/21 | Spring | Liaoning | Middle latitude | <50 |
| 168 | Discrimination of pulmonary ground-glass opacity changes in COVID‐19 and non-COVID-19 patients using CT radiomics analysis | 10.1016/j.ejro.2020.100271 | 33 | 2020/1/24 | 2020/3/31 | 2020/2/26 | Spring | Hong Kong | Low latitude | ≥50 |
| 169 | Disease Progression of Hospitalized Elderly Patients with Omicron BA.2 Treated with Molnupiravir. | 10.1007/s40121-022-00716-7 | 42 | 2020/3/26 | 2022/5/31 | 2021/4/28 | Spring | Shanghai | Middle latitude | ≥50 |
| 170 | Duration of Acute Kidney Injury and In-Hospital Mortality in Elder Patients with Severe COVID-19: A Retrospective Cohort Study | 10.1155/2022/9929038 | 107 | 2020/2/4 | 2020/4/16 | 2020/3/11 | Spring | Shanxi-陕西 | Middle latitude | ≥50 |
| 171 | Dynamic changes in clinical and CT characteristics of COVID-19 cases with different exposure histories: a retrospective study. | 10.1186/s12879-020-05306-x | 159 | 2020/1/21 | 2020/3/10 | 2020/2/14 | Spring | Beijing | Middle latitude | ≥50 |
| 172 | Dynamic evolution of COVID-19 on chest computed tomography: experience from Jiangsu Province of China. | 10.1007/s00330-020-06976-6 | 484 | 2020/1/10 | 2020/2/18 | 2020/1/29 | Winter | Jiangsu | Middle latitude | <50 |
| 173 | Dynamic profiles of SARS-Cov-2 infection from five Chinese family clusters in the early stage of the COVID-19 pandemic | 10.1038/s41598-020-79035-1 | 37 | 2020/1/28 | 2020/4/10 | 2020/3/4 | Spring | Shandong | Middle latitude | <50 |
| 174 | Dynamic trajectory of platelet-related indicators and survival of severe COVID-19 patients. | 10.1186/s13054-020-03339-x | 112 | 2019/12/1 | 2020/3/6 | 2020/1/18 | Winter | Jiangsu | Middle latitude | ≥50 |
| 175 | Early cardiac involvement in patients with acute COVID-19 infection identified by multiparametric cardiovascular magnetic resonance imaging | 10.1093/ehjci/jeab042 | 25 | 2020/3/15 | 2020/4/15 | 2020/3/30 | Spring | Shanghai | Middle latitude | <50 |
| 176 | Early chest CT features of patients with 2019 novel coronavirus (COVID-19) pneumonia: relationship to diagnosis and prognosis. | 10.1007/s00330-020-06978-4 | 34 | 2020/1/21 | 2020/2/4 | 2020/1/28 | Winter | Hainan | Low latitude | ≥50 |
| 177 | Early prediction of severity in coronavirus disease (COVID-19) using quantitative CT imaging | 10.1016/j.clinimag.2021.02.003 | 74 | 2020/1/18 | 2020/3/5 | 2020/2/10 | Spring | Guangdong | Low latitude | ≥50 |
| 178 | Early predictors of severe COVID-19 among hospitalized patients | 10.1002/jcla.24177 | 116 | 2020/1/1 | 2020/3/31 | 2020/2/15 | Spring | Henan | Middle latitude | ≥50 |
| 179 | Early therapeutic interventions of traditional Chinese medicine in COVID-19 patients: A retrospective cohort study | 10.1016/j.joim.2021.01.002 | 300 | 2020/1/26 | 2020/4/15 | 2020/3/6 | Spring | Shanghai | Middle latitude | <50 |
| 180 | Early treatment of high-risk hospitalized COVID-19 patients with a combination of interferon beta-1b and remdesivir: a phase 2 open-label randomized controlled trial. | 10.1093/cid/ciac523 | 212 | 2020/11/1 | 2021/2/28 | 2020/12/30 | Winter | Hong Kong | Low latitude | ≥50 |
| 181 | Early Viral Clearance and Antibody Kinetics of COVID-19 Among Asymptomatic Carriers. | 10.3389/fmed.2021.595773 | 75 | 2020/1/11 | 2020/4/1 | 2020/2/20 | Spring | Guangdong | Low latitude | <50 |
| 182 | Early Warning Indicators of Severe COVID-19: A Single-Center Study of Cases From Shanghai, China. | 10.3389/fmed.2020.00432 | 97 | 2020/1/20 | 2020/2/20 | 2020/2/4 | Spring | Shanghai | Middle latitude | ≥50 |
| 183 | Effect of elevated fasting blood glucose level on the 1-year mortality and sequelae in hospitalized COVID-19 patients: A bidirectional cohort study | 10.1002/jmv.27737 | 2545 | 2020/1/1 | 2020/3/18 | 2020/2/8 | Spring | Guangdong | Low latitude | ≥50 |
| 184 | Effect of nasal irrigation in adults infected with Omicron variant of COVID-19: A quasi-experimental study | 10.3389/fpubh.2022.1046112 | 80 | 2022/4/1 | 2022/5/1 | 2022/4/16 | Spring | Shandong | Middle latitude | <50 |
| 185 | Effectiveness of Chinese medicine formula Huashibaidu granule on mild COVID-19 patients: A prospective, non-randomized, controlled trial | 10.1016/j.imr.2023.100950 | 2184 | 2022/4/8 | 2022/5/6 | 2022/4/22 | Spring | Shanghai | Middle latitude | <50 |
| 186 | Effects of progressive muscle relaxation on anxiety and sleep quality in patients with COVID-19 | 10.1016/j.ctcp.2020.101132 | 26 | 2020/1/1 | 2020/2/16 | 2020/1/24 | Winter | Hainan | Low latitude | ≥50 |
| 187 | Efficacy and Safety of Leflunomide for Refractory COVID-19: A Pilot Study. | 10.3389/fphar.2021.581833 | 27 | 2020/3/13 | 2020/4/17 | 2020/3/30 | Spring | Shandong | Middle latitude | ≥50 |
| 188 | Efficacy and Safety of Lianhua Qingke Tablets in the Treatment of Mild and Common-Type COVID-19: A Randomized, Controlled, Multicenter Clinical Study. | 10.1155/2022/8733598 | 72 | 2021/1/14 | 2021/3/31 | 2021/2/21 | Spring | Hebei | Middle latitude | ≥50 |
| 189 | Efficacy and safety of ReDuNing injection as a treatment for COVID-19 and its inhibitory effect against SARS-CoV-2 | 10.1016/j.jep.2021.114367 | 50 | 2020/2/6 | 2020/3/23 | 2020/2/29 | Spring | Guangdong | Low latitude | ≥50 |
| 190 | Efficacy and safety of Reyanning mixture in patients infected with SARS-CoV-2 Omicron variant: A prospective, open-label, randomized controlled trial | 10.1016/j.phymed.2022.154514 | 2818 | 2022/4/15 | 2020/5/12 | 2021/4/28 | Spring | Shanghai | Middle latitude | <50 |
| 191 | Efficacy and Safety of Triazavirin Therapy for Coronavirus Disease 2019: A Pilot Randomized Controlled Trial | 10.1016/j.eng.2020.08.011 | 52 | 2020/2/14 | 2020/3/6 | 2020/2/24 | Spring | Heilongjiang | Middle latitude | ≥50 |
| 192 | Efficacy and safety of Xiyanping injection in the treatment of COVID-19: A multicenter, prospective, open-label and randomized controlled trial | 10.1002/ptr.7141 | 130 | 2020/1/27 | 2020/2/20 | 2020/2/8 | Spring | Jiangxi | Low latitude | <50 |
| 193 | Efficacy of Early Combination Therapy With Lianhuaqingwen and Arbidol in Moderate and Severe COVID-19 Patients: A Retrospective Cohort Study. | 10.3389/fphar.2020.560209 | 162 | 2020/1/27 | 2020/3/10 | 2020/2/17 | Spring | Shanghai | Middle latitude | ≥50 |
| 194 | Efficacy of Lianhua Qingwen for children with SARS-CoV-2 Omicron infection: A propensity score-matched retrospective cohort study | 10.1016/j.phymed.2023.154665 | 410 | 2020/4/1 | 2022/6/1 | 2021/5/1 | Spring | Shanghai | Middle latitude | <50 |
| 195 | Emotional disturbance and risk factors among COVID-19 confirmed cases in isolation hotels. | 10.1111/inm.13063 | 197 | 2020/5/28 | 2021/7/3 | 2020/12/14 | Winter | Taiwan | Low latitude | <50 |
| 196 | Epidemic characteristics of the COVID-19 outbreak in Tianjin, a well-developed city in China | 10.1016/j.ajic.2020.06.006 | 136 | 2020/1/20 | 2020/1/24 | 2020/1/22 | Winter | Tianjin | Middle latitude | <50 |
| 197 | Epidemiologic Characteristics, Transmission Chain, and Risk Factors of Severe Infection of COVID-19 in Tianjin, a Representative Municipality City of China | 10.3389/fpubh.2020.00198 | 115 | 2019/12/1 | 2020/2/20 | 2020/1/10 | Winter | Tianjin | Middle latitude | <50 |
| 198 | Epidemiological and clinical characteristics of 161 discharged cases with coronavirus disease 2019 in Shanghai, China. | 10.1186/s12879-020-05493-7 | 161 | 2019/12/1 | 2020/2/17 | 2020/1/9 | Winter | Shanghai | Middle latitude | <50 |
| 199 | Epidemiological and Clinical Characteristics of 217 Cases of COVID-19 in Jiangsu Province, China | 10.12659/MSM.930853 | 217 | 2020/1/1 | 2020/4/30 | 2020/3/1 | Spring | Jiangsu | Middle latitude | <50 |
| 200 | Epidemiological and clinical characteristics of 35 children with COVID-19 in Beijing, China. | 10.1002/ped4.12230 | 35 | 2020/1/1 | 2020/6/28 | 2020/3/30 | Spring | Beijing | Middle latitude | <50 |
| 201 | Epidemiological and clinical characteristics of COVID-19 patients in Hengyang, Hunan Province, China. | 10.12998/wjcc.v8.i12.2554 | 48 | 2020/1/16 | 2020/1/16 | 2020/1/16 | Winter | Hunan | Low latitude | <50 |
| 202 | Epidemiological and clinical characteristics of fifty-six cases of COVID-19 in Liaoning Province, China. | 10.12998/wjcc.v8.i21.5188 | 56 | 2020/1/24 | 2020/2/17 | 2020/2/5 | Spring | Liaoning | Middle latitude | <50 |
| 203 | Epidemiological and clinical characteristics of three family clusters of COVID-19 transmitted by latent patients in China. | 10.1017/S0950268820001491 | 74 | 2020/1/21 | 2020/2/9 | 2020/1/30 | Winter | Shanghai | Middle latitude | <50 |
| 204 | Epidemiological and clinical differences of coronavirus disease 2019 patients with distinct viral exposure history | 10.1080/21505594.2020.1802870 | 62 | 2020/1/20 | 2020/3/17 | 2020/2/17 | Spring | Beijing | Middle latitude | ≥50 |
| 205 | Epidemiological and clinical features in patients with coronavirus disease 2019 outside of Wuhan, China: Special focus in asymptomatic patients | 10.1371/journal.pntd.0009248 | 209 | 2020/1/1 | 2020/2/1 | 2020/1/16 | Winter | Hunan | Low latitude | <50 |
| 206 | Epidemiological and clinical features of 201 COVID-19 patients in Changsha city, Hunan, China | 10.1097/MD.0000000000021824 | 201 | 2020/1/1 | 2020/4/28 | 2020/2/29 | Spring | Hunan | Low latitude | <50 |
| 207 | Epidemiological Characteristics and Clinical Features of Patients Infected With the COVID-19 Virus in Nanchang, Jiangxi, China. | 10.3389/fmed.2020.571069 | 41 | 2020/2/4 | 2020/3/2 | 2020/2/17 | Spring | Jiangxi | Low latitude | <50 |
| 208 | Epidemiological features and dynamic changes in blood biochemical indices for COVID-19 patients in Hebi. | 10.12998/wjcc.v10.i8.2404 | 16 | 2020/1/25 | 2020/2/10 | 2020/2/2 | Winter | Henan | Middle latitude | <50 |
| 209 | Epidemiological investigation and intergenerational clinical characteristics of 24 coronavirus disease patients associated with a supermarket cluster: a retrospective study. | 10.1186/s12889-021-10713-z | 24 | 2020/1/15 | 2020/1/22 | 2020/1/18 | Winter | Shandong | Middle latitude | <50 |
| 210 | Epidemiology of 631 Cases of COVID-19 Identified in Jiangsu Province Between January 1st and March 20th 2020: Factors Associated with Disease Severity and Analysis of Zero Mortality | 10.12659/MSM.929986 | 631 | 2020/1/1 | 2020/3/20 | 2020/2/9 | Spring | Jiangsu | Middle latitude | <50 |
| 211 | Epidemiology of the first 100 cases of COVID-19 in Taiwan and its implications on outbreak control | 10.1016/j.jfma.2020.07.015 | 100 | 2020/1/11 | 2020/3/16 | 2020/2/12 | Spring | Taiwan | Low latitude | <50 |
| 212 | Epigallocatechin-3-gallate, an active ingredient of Traditional Chinese Medicines, inhibits the 3CLpro activity of SARS-CoV-2 | 10.1016/j.ijbiomac.2021.02.012 | 123 | 2020/1/19 | 2020/3/10 | 2020/2/13 | Spring | Hunan | Low latitude | <50 |
| 213 | Evaluation of chest CT and clinical features of COVID-19 patient in Macao | 10.1016/j.ejro.2020.100275 | 45 | 2020/1/22 | 2020/5/2 | 2020/3/12 | Spring | Macau | Low latitude | <50 |
| 214 | Evaluation of SARS-CoV-2 RNA shedding in clinical specimens and clinical characteristics of 10 patients with COVID-19 in Macau | 10.7150/ijbs.45357 | 10 | 2020/1/21 | 2020/2/16 | 2020/2/3 | Winter | Macau | Low latitude | ≥50 |
| 215 | Evaluation of the clinical performance of single-, dual-, and triple-target SARS-CoV-2 RT-qPCR methods | 10.1016/j.cca.2020.10.008 | 47 | 2020/1/20 | 2020/3/1 | 2020/2/9 | Spring | Jiangxi | Low latitude | <50 |
| 216 | Excessive Neutrophils and Neutrophil Extracellular Traps in COVID-19 | 10.3389/fimmu.2020.02063 | 55 | 2020/1/23 | 2020/3/15 | 2020/2/18 | Spring | Jiangsu | Middle latitude | <50 |
| 217 | Experimental Treatment with Favipiravir for COVID-19: An Open-Label Control Study | 10.1016/j.eng.2020.03.007 | 80 | 2020/1/30 | 2020/2/14 | 2020/2/6 | Spring | Guangdong | Low latitude | <50 |
| 218 | Expression of plasma IFN signaling-related miRNAs during acute SARS-CoV-2 infection and its association with RBD-IgG antibody response | 10.1186/s12985-021-01717-7 | 29 | 2020/1/1 | 2020/5/28 | 2020/3/15 | Spring | Jiangsu | Middle latitude | <50 |
| 219 | Factors associated with clinical outcomes in patients with Coronavirus Disease 2019 in Guangzhou, China | 10.1016/j.jcv.2020.104661 | 297 | 2020/1/20 | 2020/2/20 | 2020/2/4 | Spring | Guangdong | Low latitude | <50 |
| 220 | Factors associated with failure of high-flow nasal cannula oxygen therapy in patients with severe COVID-19: a retrospective case series | 10.1177/03000605221103525 | 54 | 2020/2/1 | 2020/3/26 | 2020/2/28 | Spring | Liaoning | Middle latitude | ≥50 |
| 221 | Featuring COVID-19 cases via screening symptomatic patients with epidemiologic link during flu season in a medical center of central Taiwan | 10.1016/j.jmii.2020.03.008 | 2 | 2020/1/20 | 2020/2/19 | 2020/2/4 | Spring | Taiwan | Low latitude | <50 |
| 222 | Follow-up study of clinical and chest CT scans in confirmed COVID-19 patients | 10.1016/j.jrid.2020.07.002 | 15 | 2020/1/20 | 2020/3/5 | 2020/2/11 | Spring | Guangdong | Low latitude | <50 |
| 223 | Frequency and Distribution of Chest Radiographic Findings in Patients Positive for COVID-19 | 10.1148/radiol.2020201160 | 64 | 2020/1/1 | 2020/3/28 | 2020/2/13 | Spring | Hong Kong | Low latitude | ≥50 |
| 224 | Gender Differences in Patients With COVID-19: Focus on Severity and Mortality | 10.3389/fpubh.2020.00152 | 86 | 2020/1/29 | 2020/2/15 | 2020/2/6 | Spring | Beijing | Middle latitude | ≥50 |
| 225 | Geriatric risk and protective factors for serious COVID-19 outcomes among older adults in Shanghai Omicron wave. | 10.1080/22221751.2022.2109517 | 1377 | 2022/4/1 | 2022/5/28 | 2022/4/29 | Spring | Shanghai | Middle latitude | ≥50 |
| 226 | Glycosylated hemoglobin is associated with systemic inflammation, hypercoagulability, and prognosis of COVID-19 patients | 10.1111/ijcp.14256 | 264 | 2020/2/9 | 2020/2/28 | 2020/2/18 | Spring | Beijing | Middle latitude | ≥50 |
| 227 | Hanshiyi Formula, a medicine for Sars-CoV2 infection in China, reduced the proportion of mild and moderate COVID-19 patients turning to severe status: A cohort study | 10.1016/j.phrs.2020.105127 | 1442 | 2019/12/1 | 2020/3/10 | 2020/1/20 | Winter | Beijing | Middle latitude | <50 |
| 228 | High Dose Intravenous Vitamin C for Preventing The Disease Aggravation of Moderate COVID-19 Pneumonia. A Retrospective Propensity Matched Before-After Study. | 10.3389/fphar.2021.638556 | 110 | 2020/3/18 | 2020/4/18 | 2020/4/2 | Spring | Shanghai | Middle latitude | <50 |
| 229 | HMGB1 as a potential biomarker and therapeutic target for severe COVID-19 | 10.1016/j.heliyon.2020.e05672 | 40 | 2020/1/24 | 2020/4/10 | 2020/3/2 | Spring | Hunan | Low latitude | ≥50 |
| 230 | Hypersensitivity in the lungs is responsible for acute respiratory failure in COVID-19 patients: Case series of patients who received high-dose/short-term methylprednisolone. | 10.1002/clt2.12056 | 102 | 2020/1/17 | 2020/4/26 | 2020/3/7 | Spring | Guangdong | Low latitude | <50 |
| 231 | Hypersensitivity may be involved in severe COVID-19 | 10.1111/cea.14023 | 102 | 2020/1/1 | 2020/4/25 | 2020/2/27 | Spring | Guangdong | Low latitude | <50 |
| 232 | Hypoproteinemia is an independent risk factor for the prognosis of severe COVID-19 patients. | 10.3164/jcbn.20-75 | 33 | 2020/1/20 | 2020/2/23 | 2020/2/6 | Spring | Guangdong | Low latitude | ≥50 |
| 233 | Identification of parameters in routine blood and coagulation tests related to the severity of COVID-19 | 10.7150/ijms.47494 | 311 | 2020/1/20 | 2020/3/15 | 2020/2/16 | Spring | Shanghai | Middle latitude | ≥50 |
| 234 | Identifying and quantifying robust risk factors for mortality in critically ill patients with COVID-19 using quantile regression | 10.1016/j.ajem.2020.08.090 | 192 | 2020/1/28 | 2020/3/13 | 2020/2/19 | Spring | Hunan | Low latitude | ≥50 |
| 235 | Immune Response, Viral Shedding Time, and Clinical Characterization in COVID-19 Patients With Gastrointestinal Symptoms. | 10.3389/fmed.2021.593623 | 79 | 2020/1/26 | 2020/2/16 | 2020/2/5 | Spring | Jiangsu | Middle latitude | <50 |
| 236 | Inactivated vaccine injection and immunoglobulin G levels related to severe coronavirus disease 2019 (Delta) pneumonia in Xi'an, China: A single-centered, retrospective, observational study | 10.3389/fcimb.2022.933100 | 580 | 2021/12/1 | 2022/1/31 | 2021/12/31 | Winter | Shanxi-陕西 | Middle latitude | <50 |
| 237 | Incidence, clinical course and risk factor for recurrent PCR positivity in discharged COVID-19 patients in Guangzhou, China: A prospective cohort study | 10.1371/journal.pntd.0008648 | 285 | 2020/1/20 | 2020/3/14 | 2020/2/16 | Spring | Guangdong | Low latitude | <50 |
| 238 | Increasing Age, the Existence of Comorbidities, and Corticosteroid Treatment in Combination With Antiviral Therapy Prolongs the Recovery of SARS-COV-2-Infected Patients, Measured as the Conversion From Positive to Negative rtPCR: A 239 Patients' Retrospective Study. | 10.3389/fmed.2020.575439 | 239 | 2020/1/25 | 2020/2/15 | 2020/2/4 | Spring | Hunan | Low latitude | <50 |
| 239 | Incubation period, clinical and lung CT features for early prediction of COVID-19 deterioration: development and internal verification of a risk model. | 10.1186/s12890-022-01986-0 | 239 | 2020/1/11 | 2020/2/28 | 2020/2/4 | Spring | Hunan | Low latitude | <50 |
| 240 | Indicators and prediction models for the severity of Covid-19 | 10.1111/ijcp.14571 | 98 | 2020/1/17 | 2020/2/16 | 2020/2/1 | Winter | Guangdong | Low latitude | <50 |
| 241 | Interferon-Induced Transmembrane Protein 3 Genetic Variant rs12252-C Associated With Disease Severity in Coronavirus Disease 2019 | 10.1093/infdis/jiaa224 | 80 | 2020/1/1 | 2020/2/29 | 2020/1/30 | Winter | Beijing | Middle latitude | <50 |
| 242 | Kinetic changes in virology, specific antibody response and imaging during the clinical course of COVID-19: a descriptive study. | 10.1186/s12879-020-05549-8 | 20 | 2020/1/20 | 2020/4/6 | 2020/2/27 | Spring | Shandong | Middle latitude | <50 |
| 243 | Kinetics of SARS-CoV-2 specific IgM and IgG responses in COVID-19 patients | 10.1080/22221751.2020.1762515 | 38 | 2019/12/1 | 2020/3/28 | 2020/1/29 | Winter | Guangdong | Low latitude | <50 |
| 244 | Lactate dehydrogenase and susceptibility to deterioration of mild COVID-19 patients: a multicenter nested case-control study. | 10.1186/s12916-020-01633-7 | 85 | 2020/1/17 | 2020/2/11 | 2020/1/29 | Winter | Shanghai | Middle latitude | <50 |
| 245 | Longitudinal and proteome-wide analyses of antibodies in COVID-19 patients reveal features of the humoral immune response to SARS-CoV-2 | 10.1016/j.jare.2021.07.008 | 41 | 2020/3/1 | 2020/4/30 | 2020/3/31 | Spring | Beijing | Middle latitude | <50 |
| 246 | Longitudinal changes of liver function and hepatitis B reactivation in COVID-19 patients with pre-existing chronic hepatitis B virus infection. | 10.1111/hepr.13553 | 347 | 2020/1/1 | 2020/3/1 | 2020/1/31 | Winter | Guangdong | Low latitude | <50 |
| 247 | Longitudinal trajectories of pneumonia lesions and lymphocyte counts associated with disease severity among convalescent COVID-19 patients: a group-based multi-trajectory analysis. | 10.1186/s12890-021-01592-6 | 257 | 2020/1/20 | 2020/3/31 | 2020/2/24 | Spring | Shanghai | Middle latitude | ≥50 |
| 248 | Low high-density lipoprotein level is correlated with the severity of COVID-19 patients: an observational study | 10.1186/s12944-020-01382-9 | 228 | 2020/1/17 | 2020/3/14 | 2020/2/14 | Spring | Hunan | Low latitude | <50 |
| 249 | Low-to-moderate dose corticosteroids treatment in hospitalized adults with COVID-19 | 10.1016/j.cmi.2020.09.045 | 488 | 2019/12/29 | 2020/2/15 | 2020/1/22 | Winter | Beijing | Middle latitude | ≥50 |
| 250 | Lymphocyte blood levels that remain low can predict the death of patients with COVID-19. | 10.1097/MD.0000000000026503 | 205 | 2020/2/10 | 2020/3/8 | 2020/2/23 | Spring | Shanxi-陕西 | Middle latitude | ≥50 |
| 251 | Lymphocyte percentage and hemoglobin as a joint parameter for the prediction of severe and nonsevere COVID-19: a preliminary study. | 10.21037/atm-20-6001 | 159 | 2020/1/23 | 2020/3/21 | 2020/2/21 | Spring | Guangdong | Low latitude | ≥50 |
| 252 | Lymphocyte subset alterations with disease severity, imaging manifestation, and delayed hospitalization in COVID-19 patients. | 10.1186/s12879-021-06354-7 | 106 | 2020/2/23 | 2020/2/29 | 2020/2/26 | Spring | Jiangxi | Low latitude | <50 |
| 253 | Lymphopenia predicted illness severity and recovery in patients with COVID-19: A single-center, retrospective study | 10.1371/journal.pone.0241659 | 230 | 2020/1/7 | 2020/2/14 | 2020/1/26 | Winter | Hunan | Low latitude | <50 |
| 254 | Lysosome activation in peripheral blood mononuclear cells and prognostic significance of circulating LC3B in COVID-19 | 10.1093/bib/bbab043 | 168 | 2020/1/2 | 2020/3/8 | 2020/2/4 | Spring | Guangdong | Low latitude | <50 |
| 255 | Machine learning based on clinical characteristics and chest CT quantitative measurements for prediction of adverse clinical outcomes in hospitalized patients with COVID-19. | 10.1007/s00330-021-07957-z | 424 | 2020/1/17 | 2020/2/17 | 2020/2/1 | Winter | Hunan | Low latitude | ≥50 |
| 256 | Machine learning-based CT radiomics model distinguishes COVID-19 from non-COVID-19 pneumonia | 10.1186/s12879-021-06614-6 | 63 | 2020/1/20 | 2020/2/8 | 2020/1/29 | Winter | Hainan | Low latitude | ≥50 |
| 257 | Medical treatment of 55 patients with COVID-19 from seven cities in northeast China who fully recovered A single-center, retrospective, observational study | 10.1097/MD.0000000000023923 | 55 | 2020/1/20 | 2020/3/15 | 2020/2/16 | Spring | Liaoning | Middle latitude | <50 |
| 258 | Metabolomic analyses reveal new stage-specific features of COVID-19 | 10.1183/13993003.00284-2021 | 50 | 2020/1/1 | 2020/3/31 | 2020/2/15 | Spring | Guangdong | Low latitude | ≥50 |
| 259 | Metagenomic Next-Generation Sequencing Reveals the Profile of Viral Infections in Kidney Transplant Recipients During the COVID-19 Pandemic. | 10.3389/fpubh.2022.888064 | 39 | 2020/5/1 | 2021/5/28 | 2020/11/13 | Winter | Henan | Middle latitude | <50 |
| 260 | Mild Cytokine Elevation, Moderate CD4(+) T Cell Response and Abundant Antibody Production in Children with COVID-19 | 10.1007/s12250-020-00265-8 | 19 | 2020/1/19 | 2020/4/8 | 2020/2/28 | Spring | Shanghai | Middle latitude | ≥50 |
| 261 | Milder symptoms and shorter course in patients with re-positive COVID-19: A cohort of 180 patients from Northeast China. | 10.3389/fmicb.2022.989879 | 360 | 2020/3/1 | 2020/6/30 | 2020/4/30 | Spring | Jilin | Middle latitude | <50 |
| 262 | Moderate vs. mild cases of overseas-imported COVID-19 in Beijing: a retrospective cohort study | 10.1038/s41598-021-85869-0 | 53 | 2020/3/16 | 2020/4/30 | 2020/4/7 | Spring | Beijing | Middle latitude | <50 |
| 263 | Multicenter evaluation of two chemiluminescence and three lateral flow immunoassays for the diagnosis of COVID-19 and assessment of antibody dynamic responses to SARS-CoV-2 in Taiwan | 10.1080/22221751.2020.1825016 | 74 | 2020/1/23 | 2020/5/31 | 2020/3/27 | Spring | Taiwan | Low latitude | <50 |
| 264 | Myocardial Injury on Admission as a Risk in Critically Ill COVID-19 Patients: A Retrospective in-ICU Study | 10.1053/j.jvca.2020.10.019 | 36 | 2020/2/4 | 2020/3/3 | 2020/2/18 | Spring | Beijing | Middle latitude | ≥50 |
| 265 | Myosteatosis predicting risk of transition to severe COVID-19 infection | 10.1016/j.clnu.2021.05.031 | 234 | 2020/1/21 | 2020/2/19 | 2020/2/4 | Spring | Hunan | Low latitude | <50 |
| 266 | Neurosensory dysfunction: A diagnostic marker of early COVID-19 | 10.1016/j.ijid.2020.06.086 | 86 | 2020/3/16 | 2020/4/12 | 2020/3/29 | Spring | Guangdong | Low latitude | <50 |
| 267 | Olfactory disorder in patients infected with SARS-CoV-2 | 10.1016/j.jmii.2020.08.010BY- | 5 | 2020/3/22 | 2020/4/3 | 2020/3/28 | Spring | Taiwan | Low latitude | <50 |
| 268 | Oral vitamin D supplemental therapy to attain a desired serum 25-hydroxyvitamin D concentration in essential healthcare teams | 10.1186/s13063-022-06944-z | 24 | 2020/1/15 | 2020/1/22 | 2020/1/18 | Winter | Shandong | Middle latitude | <50 |
| 269 | Original Article Out-of-hospital cardiac arrest and in-hospital mortality among COVID-19 patients: A population-based retrospective cohort study | 10.1016/j.jmii.2022.07.009 | 2555 | 2020/5/15 | 2021/6/20 | 2020/12/1 | Winter | Taiwan | Low latitude | ≥50 |
| 270 | Overweight and Obesity are Risk Factors of Severe Illness in Patients with COVID-19. | 10.1002/oby.22979 | 297 | 2020/1/18 | 2020/2/26 | 2020/2/6 | Spring | Jiangsu | Middle latitude | <50 |
| 271 | Patterns of Deterioration in Moderate Patients With COVID-19 From Jan 2020 to Mar 2020: A Multi-Center, Retrospective Cohort Study in China. | 10.3389/fmed.2020.567296 | 1168 | 2020/1/14 | 2020/3/16 | 2020/2/14 | Spring | Guangdong | Low latitude | <50 |
| 272 | Plasma IP-10 and MCP-3 levels are highly associated with disease severity and predict the progression of COVID-19 | 10.1016/j.jaci.2020.04.027 | 50 | 2019/12/1 | 2020/3/31 | 2020/1/30 | Winter | Guangdong | Low latitude | ≥50 |
| 273 | Positive RT-PCR tests among discharged COVID-19 patients in Shenzhen, China. | 10.1017/ice.2020.134 | 583 | 2020/1/23 | 2020/2/21 | 2020/2/6 | Spring | Guangdong | Low latitude | <50 |
| 274 | Predicting Illness Severity and Short-Term Outcomes of COVID-19: A Retrospective Cohort Study in China | 10.1016/j.xinn.2020.04.007 | 417 | 2020/1/11 | 2020/2/18 | 2020/1/30 | Winter | Guangdong | Low latitude | <50 |
| 275 | Prediction of disease progression in patients with COVID-19 by artificial intelligence assisted lesion quantification | 10.1038/s41598-020-79097-1 | 246 | 2020/2/10 | 2020/4/9 | 2020/3/10 | Spring | Shanghai | Middle latitude | ≥50 |
| 276 | Predictors of clinical deterioration in non-severe patients with COVID-19: a retrospective cohort study | 10.1080/03007995.2021.1876005 | 257 | 2020/1/23 | 2020/3/21 | 2020/2/21 | Spring | Guangdong | Low latitude | <50 |
| 277 | Predictors of COVID-19 Infection: A Prevalence Study of Hospitalized Patients. | 10.1155/2021/6213450 | 96 | 2020/1/19 | 2020/2/18 | 2020/2/3 | Winter | Shanxi-陕西 | Middle latitude | ≥50 |
| 278 | Pre-existing liver disease is associated with poor outcome in patients with SARS CoV2 infection; The APCOLIS Study (APASL COVID-19 Liver Injury Spectrum Study). | 10.1007/s12072-020-10072-8 | 271 | 2020/1/1 | 2020/4/1 | 2020/2/15 | Spring | Shanghai | Middle latitude | <50 |
| 279 | Preliminary evidence from a multicenter prospective observational study of the safety and efficacy of chloroquine for the treatment of COVID-19. | 10.1093/nsr/nwaa113 | 373 | 2020/2/7 | 2020/3/8 | 2020/2/22 | Spring | Guangdong | Low latitude | <50 |
| 280 | Preliminary Exploration of the Cause of Liver Disorders During Early Stages in COVID-19 Patients. | 10.3389/fmed.2020.00501 | 44 | 2020/1/21 | 2020/2/24 | 2020/2/7 | Spring | Beijing | Middle latitude | ≥50 |
| 281 | Preliminary investigation of relationship between clinical indicators and CT manifestation patterns of COVID-19 pneumonia improvement. | 10.21037/jtd-20-1420 | 62 | 2020/1/20 | 2020/2/8 | 2020/1/29 | Winter | Shanghai | Middle latitude | <50 |
| 282 | Prevalence and outcomes of re-positive nucleic acid tests in discharged COVID-19 patients | 10.1007/s10096-020-04024-1 | 3 | 2020/2/15 | 2020/3/14 | 2020/2/29 | Spring | Fujian | Low latitude | ≥50 |
| 283 | Prevalence of taste and smell dysfunction in mild and asymptomatic COVID-19 patients during Omicron prevalent period in Shanghai, China: a cross-sectional survey study. | 10.1136/bmjopen-2022-067065 | 2058 | 2020/3/1 | 2022/4/28 | 2021/3/30 | Spring | Shanghai | Middle latitude | <50 |
| 284 | Prognostic Accuracy of Early Warning Scores for Clinical Deterioration in Patients With COVID-19. | 10.3389/fmed.2020.624255 | 116 | 2020/2/7 | 2020/2/17 | 2020/2/12 | Spring | Shanghai | Middle latitude | ≥50 |
| 285 | Prognostic Factors for COVID-19 Pneumonia Progression to Severe Symptoms Based on Earlier Clinical Features: A Retrospective Analysis. | 10.3389/fmed.2020.557453 | 125 | 2020/1/20 | 2020/2/29 | 2020/2/9 | Spring | Guangdong | Low latitude | <50 |
| 286 | Prognostic value of interleukin-6, C-reactive protein, and procalcitonin in patients with COVID-19 | 10.1016/j.jcv.2020.104370 | 140 | 2020/1/18 | 2020/3/12 | 2020/2/14 | Spring | Beijing | Middle latitude | ≥50 |
| 287 | Proteomic profiling reveals a distinctive molecular signature for critically ill COVID-19 patients compared with asthma and chronic obstructive pulmonary disease | 10.1016/j.ijid.2022.01.008 | 5 | 2020/2/26 | 2020/2/15 | 2020/2/20 | Spring | Guangdong | Low latitude | ≥50 |
| 288 | Psychological distress assessment among patients with suspected and confirmed COVID-19: A cohort study | 10.1016/j.jfma.2021.02.014 | 109 | 2020/1/1 | 2020/5/31 | 2020/3/16 | Spring | Taiwan | Low latitude | <50 |
| 289 | Pulmonary fibrosis and its related factors in discharged patients with new corona virus pneumonia: a cohort study | 10.1186/s12931-021-01798-6 | 289 | 2020/1/11 | 2020/4/26 | 2020/3/4 | Spring | Guangdong | Low latitude | <50 |
| 290 | Pulmonary fibrosis in patients with COVID-19: A retrospective study | 10.3389/fcimb.2022.1013526 | 227 | 2019/12/31 | 2020/3/26 | 2020/2/12 | Spring | Hunan | Low latitude | <50 |
| 291 | Qingjin Yiqi granules for post-COVID-19 condition: A randomized clinical trial | 10.1111/jebm.12465 | 388 | 2021/1/1 | 2021/3/31 | 2021/2/14 | Spring | Beijing | Middle latitude | <50 |
| 292 | Quantitative computed tomography of the coronavirus disease 2019 (COVID-19) pneumonia | 10.1016/j.jrid.2020.04.004 | 30 | 2020/1/17 | 2020/2/9 | 2020/1/28 | Winter | Shanghai | Middle latitude | <50 |
| 293 | Quantitative Detection and Viral Load Analysis of SARS-CoV-2 in Infected Patients | 10.1093/cid/ciaa345 | 76 | 2020/2/5 | 2020/2/19 | 2020/2/12 | Spring | Beijing | Middle latitude | <50 |
| 294 | Radiomics nomogram for the prediction of 2019 novel coronavirus pneumonia caused by SARS-CoV-2. | 10.1007/s00330-020-07032-z | 192 | 2020/2/11 | 2020/2/25 | 2020/2/18 | Spring | Shanghai | Middle latitude | ≥50 |
| 295 | Reactivation of SARS-CoV-2 infection following recovery from COVID-19 | 10.1016/j.jiph.2021.02.002 | 109 | 2020/1/18 | 2020/4/23 | 2020/3/6 | Spring | Beijing | Middle latitude | <50 |
| 296 | Relationship between blood eosinophil levels and COVID-19 mortality | 10.1016/j.waojou.2021.100521 | 190 | 2020/1/28 | 2020/3/25 | 2020/2/25 | Spring | Jilin | Middle latitude | ≥50 |
| 297 | Research on Influencing Factors and Classification of Patients With Mild and Severe COVID-19 Symptoms | 10.3389/fcimb.2021.670823 | 90 | 2020/1/21 | 2020/3/31 | 2020/2/25 | Spring | Guangdong | Low latitude | <50 |
| 298 | Respiratory failure among patients with COVID-19 in Jiangsu province, China: a multicentre retrospective cohort study | 10.1017/S0950268821000157 | 625 | 2020/1/10 | 2020/3/15 | 2020/2/11 | Spring | Jiangsu | Middle latitude | <50 |
| 299 | Retrospective cohort study comparing the epidemiological and clinical characteristics between imported and local COVID-19 inpatients in Nanyang, China. | 10.1136/jim-2020-001643 | 129 | 2020/1/24 | 2020/2/26 | 2020/2/9 | Spring | Hebei | Middle latitude | <50 |
| 300 | Reyanning Mixture on Asymptomatic or Mild SARS-CoV-2 Infection in Children and Adolescents:A Randomized Controlled Trial | 10.1007/s11655-023-3609-0 | 866 | 2022/4/15 | 2022/5/12 | 2022/4/28 | Spring | Shanghai | Middle latitude | ≥50 |
| 301 | Rheumatic Symptoms Following Coronavirus Disease 2019 (COVID-19): A Chronic Post-COVID-19 Condition. | 10.1093/ofid/ofac170 | 1296 | 2020/12/16 | 2021/2/7 | 2021/1/11 | Winter | Beijing | Middle latitude | ≥50 |
| 302 | Ribavirin Treatment for Critically Ill COVID-19 Patients: An Observational Study. | 10.2147/IDR.S330743 | 19 | 2020/1/27 | 2020/4/18 | 2020/3/8 | Spring | Guangdong | Low latitude | ≥50 |
| 303 | Risk factors for disease progression in hospitalized patients with COVID-19: a retrospective cohort study | 10.1080/23744235.2020.1759817 | 101 | 2020/1/21 | 2020/3/9 | 2020/2/14 | Spring | Beijing | Middle latitude | ≥50 |
| 304 | Risk factors for severe cases of COVID-19: a retrospective cohort study | 10.3390/ijms22147462 | 288 | 2020/1/15 | 2020/3/10 | 2020/2/11 | Spring | Guangdong | Low latitude | <50 |
| 305 | Risk factors for severe COVID-19 in middle-aged patients without comorbidities: a multicentre retrospective study. | 10.1186/s12967-020-02655-8 | 119 | 2020/1/31 | 2020/4/17 | 2020/3/9 | Spring | Shandong | Middle latitude | ≥50 |
| 306 | Risk factors for the COVID-19 severity and its correlation with viral shedding: A retrospective cohort study | 10.1002/jmv.26367 | 80 | 2020/1/17 | 2020/3/9 | 2020/2/12 | Spring | Guangdong | Low latitude | ≥50 |
| 307 | Risk of severe illness of COVID-19 patients with NAFLD and increased NAFLD fibrosis scores | 10.1002/jcla.23880 | 48 | 2020/1/18 | 2020/2/26 | 2020/2/6 | Spring | Jiangsu | Middle latitude | <50 |
| 308 | Safety and Efficacy of Paxlovid Against Omicron Variants of Coronavirus Disease 2019 in Elderly Patients | 10.1007/s40121-023-00760-x | 163 | 2022/4/1 | 2022/5/31 | 2022/5/1 | Spring | Shanghai | Middle latitude | ≥50 |
| 309 | Serum triglyceride levels and related factors as prognostic indicators in COVID-19 patients: A retrospective study | 10.1002/iid3.469 | 174 | 2020/2/9 | 2020/2/29 | 2020/2/19 | Spring | Beijing | Middle latitude | ≥50 |
| 310 | Settings of virus exposure and their implications in the propagation of transmission networks in a COVID-19 outbreak. | 10.1016/j.lanwpc.2020.100052 | 450 | 2020/1/23 | 2020/6/1 | 2020/3/28 | Spring | Hong Kong | Low latitude | <50 |
| 311 | Severe Acute Respiratory Syndrome Coronavirus 2 Viral RNA Load Status and Antibody Distribution Among Patients and Asymptomatic Carriers in Central China | 10.3389/fcimb.2021.559447 | 65 | 2020/1/2 | 2020/4/29 | 2020/3/1 | Spring | Henan | Middle latitude | ≥50 |
| 312 | Sex differences in clinical characteristics and risk factors for disease severity of hospitalized patients with COVID-19. | 10.1002/mco2.66 | 185 | 2019/11/1 | 2020/3/19 | 2020/1/9 | Winter | Beijing | Middle latitude | <50 |
| 313 | Sex Differences on Clinical Characteristics, Severity, and Mortality in Adult Patients With COVID-19: A Multicentre Retrospective Study. | 10.3389/fmed.2021.607059 | 413 | 2020/1/31 | 2020/4/17 | 2020/3/9 | Spring | Shandong | Middle latitude | ≥50 |
| 314 | Shorter incubation period is associated with severe disease progression in patients with COVID-19 | 10.1080/21505594.2020.1836894 | 330 | 2020/1/11 | 2020/2/10 | 2020/1/26 | Winter | Guangdong | Low latitude | <50 |
| 315 | Smoking Is Correlated With the Prognosis of Coronavirus Disease 2019 (COVID-19) Patients: An Observational Study. | 10.3389/fphys.2021.634842 | 622 | 2020/1/1 | 2020/3/26 | 2020/2/12 | Spring | Hunan | Low latitude | <50 |
| 316 | Specific dynamic variations in the peripheral blood lymphocyte subsets in COVID-19 and severe influenza A patients: a retrospective observational study. | 10.1186/s12879-020-05637-9 | 99 | 2020/1/20 | 2020/3/17 | 2020/2/17 | Spring | Beijing | Middle latitude | <50 |
| 317 | Survivors of COVID-19 exhibit altered amplitudes of low frequency fluctuation in the brain: a resting-state functional magnetic resonance imaging study at 1-year follow-up. | 10.4103/1673-5374.327361 | 19 | 2019/12/1 | 2020/3/19 | 2020/1/24 | Winter | Hunan | Low latitude | ≥50 |
| 318 | T-Cell Repertoire Characteristics of Asymptomatic and Re-Detectable Positive COVID-19 Patients | 10.3389/fimmu.2021.769442 | 54 | 2020/3/1 | 2020/5/31 | 2020/4/15 | Spring | Guangdong | Low latitude | <50 |
| 319 | Temporal landscape of human gut RNA and DNA virome in SARS-CoV-2 infection and severity | 10.1186/s40168-021-01008-x | 98 | 2020/2/1 | 2020/5/28 | 2020/3/30 | Spring | Hong Kong | Low latitude | <50 |
| 320 | Temporal lung changes on thin-section CT in patients with COVID-19 pneumonia. | 10.1038/s41598-020-76776-x | 56 | 2020/1/22 | 2020/3/3 | 2020/2/11 | Spring | Guangdong | Low latitude | <50 |
| 321 | Temporal profiles of viral load in posterior oropharyngeal saliva samples and serum antibody responses during infection by SARS-CoV-2: an observational cohort study | 10.1016/S1473-3099(20)30196-1 | 23 | 2020/1/22 | 2020/2/12 | 2020/2/1 | Winter | Hong Kong | Low latitude | ≥50 |
| 322 | Texture feature-based machine learning classifier could assist in the diagnosis of COVID-19 | 10.1016/j.ejrad.2021.109602 | 191 | 2019/12/30 | 2020/3/30 | 2020/2/13 | Spring | Beijing | Middle latitude | ≥50 |
| 323 | The Clinical Features and Prognostic Assessment of SARS-CoV-2 Infection-Induced Sepsis Among COVID-19 Patients in Shenzhen, China. | 10.3389/fmed.2020.570853 | 747 | 2020/1/11 | 2020/4/27 | 2020/3/4 | Spring | Guangdong | Low latitude | <50 |
| 324 | The clinical implication of dynamic neutrophil to lymphocyte ratio and D-dimer in COVID-19: A retrospective study in Suzhou China | 10.1016/j.thromres.2020.05.006 | 75 | 2020/1/20 | 2020/2/20 | 2020/2/4 | Spring | Jiangsu | Middle latitude | <50 |
| 325 | The clinical value of bedside ultrasound in predicting the severity of coronavirus disease-19 (COVID-19). | 10.21037/atm-20-7944 | 31 | 2020/1/18 | 2020/2/5 | 2020/1/27 | Winter | Guangdong | Low latitude | ≥50 |
| 326 | The differences of clinical characteristics and outcomes between imported and local patients of COVID-19 in Hunan: a two-center retrospective study. | 10.1186/s12931-020-01551-5 | 169 | 2020/1/21 | 2020/2/21 | 2020/2/5 | Spring | Hunan | Low latitude | <50 |
| 327 | The effects of renin-angiotensin system inhibitors (RASI) in coronavirus disease (COVID-19) with hypertension: A retrospective, single-center trial | 10.1016/j.medcli.2020.06.007 | 13 | 2020/1/25 | 2020/1/31 | 2020/1/28 | Winter | Shanghai | Middle latitude | ≥50 |
| 328 | The effects of vaccination on the disease severity and factors for viral clearance and hospitalization in Omicron-infected patients: A retrospective observational cohort study from recent regional outbreaks in China. | 10.3389/fcimb.2022.988694 | 3491 | 2022/3/13 | 2021/10/20 | 2021/12/31 | Winter | Fujian | Low latitude | <50 |
| 329 | The epidemiologic and clinical features of suspected and confirmed cases of imported 2019 novel coronavirus pneumonia in north Shanghai, China. | 10.21037/atm-20-2119 | 21 | 2020/1/1 | 2020/2/15 | 2020/1/23 | Winter | Shanghai | Middle latitude | ≥50 |
| 330 | The feasibility, safety, and efficacy of Paxlovid treatment in SARS-CoV-2-infected children aged 6-14 years: a cohort study. | 10.21037/atm-22-2791 | 30 | 2022/4/7 | 2022/5/26 | 2022/5/1 | Spring | Shanghai | Middle latitude | <50 |
| 331 | The impact of COVID-19 on gastric cancer surgery: a single-center retrospective study. | 10.1186/s12893-020-00885-7 | 41 | 2019/12/20 | 2020/3/20 | 2020/2/3 | Winter | Beijing | Middle latitude | ≥50 |
| 332 | The implications of preliminary screening and diagnosis: Clinical characteristics of 33 mild patients with SARS-CoV-2 infection in Hunan, China | 10.1097/MD.0000000000029920 | 74 | 2020/1/19 | 2020/2/7 | 2020/1/28 | Winter | Hunan | Low latitude | <50 |
| 333 | The Independent Association of TSH and Free Triiodothyronine Levels With Lymphocyte Counts Among COVID-19 Patients | 10.3389/fendo.2021.774346 | 541 | 2020/7/21 | 2021/4/20 | 2020/12/4 | Winter | Hong Kong | Low latitude | ≥50 |
| 334 | The occurrence of and risk factors for developing acute critical illness during quarantine as a response to the COVID-19 pandemic | 10.1016/j.jfma.2021.01.013 | 320 | 2020/3/1 | 2020/5/31 | 2020/4/15 | Spring | Taiwan | Low latitude | ≥50 |
| 335 | The Performance of Chest CT in Evaluating the Clinical Severity of COVID-19 Pneumonia: Identifying Critical Cases Based on CT Characteristics | 10.1097/RLI.0000000000000689 | 51 | 2020/1/15 | 2020/2/24 | 2020/2/4 | Spring | Henan | Middle latitude | ≥50 |
| 336 | The Predictive Effectiveness of Blood Biochemical Indexes for the Severity of COVID-19. | 10.1155/2020/7320813 | 108 | 2020/1/30 | 2020/2/19 | 2020/2/9 | Spring | Hunan | Low latitude | <50 |
| 337 | The predictive value of serum level of cystatin C for COVID-19 severity | 10.1038/s41598-021-01570-2 | 162 | 2020/2/15 | 2020/3/14 | 2020/2/29 | Spring | Fujian | Low latitude | ≥50 |
| 338 | The preliminary comparative results between Covid-19 and non-Covid-19 patients in Western China. | 10.1186/s12879-020-05680-6 | 15 | 2020/1/20 | 2020/2/26 | 2020/2/7 | Spring | Shanxi-陕西 | Middle latitude | <50 |
| 339 | The Relationship Between Chest Imaging Findings and the Viral Load of COVID-19. | 10.3389/fmed.2020.558539 | 56 | 2020/1/16 | 2020/2/6 | 2020/1/26 | Winter | Hunan | Low latitude | ≥50 |
| 340 | The role of peripheral blood eosinophil counts in COVID-19 patients | 10.1111/all.14465 | 212 | 2020/1/22 | 2020/2/6 | 2020/1/29 | Winter | Shanghai | Middle latitude | ≥50 |
| 341 | The timeline and risk factors of clinical progression of COVID-19 in Shenzhen, China. | 10.1186/s12967-020-02423-8 | 323 | 2020/1/10 | 2020/2/10 | 2020/1/25 | Winter | Guangdong | Low latitude | <50 |
| 342 | The underlying changes and predicting role of peripheral blood inflammatory cells in severe COVID-19 patients: A sentinel? | 10.1016/j.cca.2020.05.027 | 57 | 2020/2/10 | 2020/3/8 | 2020/2/23 | Spring | Jilin | Middle latitude | ≥50 |
| 343 | The value of longitudinal clinical data and paired CT scans in predicting the deterioration of COVID-19 revealed by an artificial intelligence system | 10.1016/j.isci.2022.104227 | 119 | 2020/1/21 | 2020/4/29 | 2020/3/10 | Spring | Shanghai | Middle latitude | ≥50 |
| 344 | The value of repeated CT in monitoring the disease progression in moderate COVID-19 pneumonia A single-center, retrospective study | 10.1097/MD.0000000000025005 | 43 | 2020/1/22 | 2020/2/7 | 2020/1/30 | Winter | Fujian | Low latitude | <50 |
| 345 | The value of the wireless stethoscope in patients with COVID-19 infection in a makeshift hospital | 10.1186/s12938-023-01136-5 | 400 | 2022/4/10 | 2022/5/10 | 2022/4/25 | Spring | Shanghai | Middle latitude | <50 |
| 346 | Transcriptome Analysis of Peripheral Blood Mononuclear Cells Reveals Distinct Immune Response in Asymptomatic and Re-Detectable Positive COVID-19 Patients | 10.3389/fimmu.2021.716075 | 48 | 2020/3/1 | 2020/5/31 | 2020/4/15 | Spring | Guangdong | Low latitude | <50 |
| 347 | Transmission and clinical characteristics of coronavirus disease 2019 in 104 outside-Wuhan patients, China | 10.1002/jmv.25975 | 208 | 2020/1/22 | 2020/2/12 | 2020/2/1 | Winter | Hunan | Low latitude | <50 |
| 348 | Transmission of SARS-CoV-2 during air travel: a descriptive and modelling study | 10.1080/07853890.2021.1973084 | 161 | 2020/3/1 | 2020/3/31 | 2020/3/16 | Spring | Beijing | Middle latitude | <50 |
| 349 | Understanding clinical characteristics influencing adverse outcomes of Omicron infection: a retrospective study with propensity score matching from a Fangcang hospital. | 10.3389/fcimb.2023.1115089 | 390 | 2020/4/6 | 2022/5/16 | 2021/4/26 | Spring | Jiangsu | Middle latitude | ≥50 |
| 350 | Unvaccinated Children Are an Important Link in the Transmission of SARS-CoV-2 Delta Variant (B1.617.2): Comparative Clinical Evidence From a Recent Community Surge | 10.3389/fcimb.2022.814782 | 452 | 2020/9/10 | 2021/10/20 | 2021/3/31 | Spring | Fujian | Low latitude | <50 |
| 351 | Vitamin D levels and clinical outcomes of SARS-CoV-2 Omicron subvariant BA.2 in children: A longitudinal cohort study. | 10.3389/fnut.2022.960859 | 116 | 2019/12/1 | 2022/4/1 | 2021/1/30 | Winter | Guangdong | Low latitude | ≥50 |
| 352 | 从“湿热疫毒”诊治24例新型冠状病毒肺炎的临床研究及体会 | 10.16367/j.issn.1003-5028.2020.05.0167 | 46 | 2020/2/3 | 2020/2/18 | 2020/2/10 | Spring | Beijing | Middle latitude | ≥50 |
| 353 | 高血压对COVID-19患者临床结局的影响 | 10.12122/j.issn.1673-4254.2020.11.01 | 64 | 2020/1/1 | 2020/3/18 | 2020/2/8 | Spring | Guangdong | Low latitude | ≥50 |
| 354 | 广西144例家族聚集性SARS-CoV-2 Omicron变异株感染者的临床特征分析 | 10.1111/ajt.16251 | 144 | 2022/2/4 | 2022/3/2 | 2022/2/17 | Spring | Guangxi | Low latitude | <50 |
| 355 | 广州29例境外输入型新型冠状病毒肺炎的临床特点分析 | 10.13406/j.cnki.cyxb.002517 | 44 | 2020/3/11 | 2020/3/21 | 2020/3/16 | Spring | Guangdong | Low latitude | ≥50 |
| 356 | 河南省新型冠状病毒肺炎流行期疫情特征分析 | 10.13705/j.issn.1671-6825.2020.03.002 | 2474 | 2019/12/1 | 2020/2/19 | 2020/1/10 | Winter | Henan | Middle latitude | <50 |
| 357 | 基于实验室指标建立的COVID-19与非COVID-19患者鉴别诊断模型 | 10.13820/j.cnki.gdyx.20212516 | 132 | 2020/1/1 | 2020/4/28 | 2020/2/29 | Spring | Jiangxi | Low latitude | <50 |
| 358 | 天津地区新型冠状病毒肺炎患者临床特征分析 | 10.11958/20201093 | 135 | 2020/1/21 | 2020/3/15 | 2020/2/17 | Spring | Tianjin | Middle latitude | <50 |
| 359 | 新冠肺炎患者焦虑症状及相关因素 | 10.13192/j.issn.1000-1719.2021.11.028 | 108 | 2020/2/1 | 2020/2/28 | 2020/2/14 | Spring | Hunan | Low latitude | <50 |
| 360 | 新型冠状病毒肺炎患者的临床特征及护理 | 10.3969/j.issn.2095-5227.2020.03.003 | 61 | 2020/1/20 | 2020/2/29 | 2020/2/9 | Spring | Beijing | Middle latitude | <50 |
| 361 | 新型冠状病毒肺炎疫情期间发热门诊重症患者的临床特征 | 10.11816/cn.ni.2020-201163 | 80 | 2020/2/29 | 2020/3/31 | 2020/3/15 | Spring | Beijing | Middle latitude | ≥50 |
| 362 | 信阳市新型冠状病毒肺炎重型/危重型早期危险因素筛选研究 | 10.14188/j.1671-8852.2020.0537 | 220 | 2020/1/22 | 2020/2/28 | 2020/2/9 | Spring | Henan | Middle latitude | ≥50 |
| 363 | 中性粒细胞/淋巴细胞比值可作为重型COVID-19的预警信号 | 10.12122/j.issn.1673-4254.2020.03.06 | 126 | 2020/1/26 | 2020/2/20 | 2020/2/7 | Spring | Guangdong | Low latitude | ≥50 |
| 364 | 重型与非重型新型冠状病毒肺炎患者临床特征及预后的差异分析 | 10.3969/j.issn.2095-5227.2020.07.001 | 95 | 2020/1/21 | 2020/2/24 | 2020/2/7 | Spring | Beijing | Middle latitude | ≥50 |
| 365 | 重症2019冠状病毒病早期临床特征及重症化危险因素分析 | 10.1182/bloodadvances.2020003513 | 21 | 2020/1/20 | 2020/3/3 | 2020/2/10 | Spring | Beijing | Middle latitude | ≥50 |
| 366 | 重症新型冠状病毒肺炎患者的临床特征及预后风险因素分析 | 10.13718/j.cnki.xdzk.2020.03.003 | 1276 | 2020/2/4 | 2020/4/10 | 2020/3/8 | Spring | Beijing | Middle latitude | ≥50 |
